# Multiple data revealed two new species of the Asian horned toad *Megophrys* Kuhl & Van Hasselt, 1822 (Anura, Megophryidae) from the eastern corner of the Himalayas

**DOI:** 10.3897/zookeys.977.55693

**Published:** 2020-10-22

**Authors:** Shengchao Shi, Meihua Zhang, Feng Xie, Jianping Jiang, Wulin Liu, Li Ding, Li Luan, Bin Wang

**Affiliations:** 1 CAS Key Laboratory of Mountain Ecological Restoration and Bioresource Utilization & Ecological Restoration Biodiversity Conservation Key Laboratory of Sichuan Province, Chengdu Institute of Biology, Chinese Academy of Sciences, Chengdu 610041, China Chinese Academy of Sciences Chengdu China; 2 Key Laboratory of Bio-Resource and Eco-Environment of Ministry of Education, College of Life Sciences, Sichuan University, Chengdu 610065, Sichuan, China Sichuan University Chengdu China; 3 University of Chinese Academy of Sciences, Beijing 100049, China University of Chinese Academy of Sciences Beijing China; 4 Forestry Survey and Design Research Institute of the Tibet Autonomous Region, Lhasa 850000, China Forestry Survey and Design Research Institute of the Tibet Autonomous Region Lhasa China; 5 Chengdu Survey and Design Research Institute of China Electric Power Construction Group Co., Ltd., Chengdu 610041, China Chengdu Survey and Design Research Institute of China Electric Power Construction Group Co., Ltd. Chengdu China

**Keywords:** Multiple data, taxonomy, molecular phylogenetic analyses, morphology, introgression

## Abstract

Multiple disciplines can help to discover cryptic species and resolve taxonomic confusions. The Asian horned toad genus *Megophryssensu lato* as a diverse group was proposed to contain dozens of cryptic species. Based on molecular phylogenetics, morphology, osteology, and bioacoustics data, the species profiles of *Megophrys* toads in the eastern corner of Himalayas in Medog County, Tibet Autonomous Region, China was investigated. The results indicated that this small area harbored at least four *Megophrys* species, i.e., *M. medogensis*, *M. pachyproctus*, *Megophrys zhoui***sp. nov.**, and *Megophrys yeae***sp. nov.**, the latter two being described in this study. Additionally, the mitochondrial DNA trees nested the low-middle-elevation and high-elevation groups of *M. medogensis* into a monophyletic group, being in discordance with the paraphyletic relationship between them revealed in the nuclear DNA trees. The findings highlighted the underestimated biodiversity in Himalayas, and further indicated that the *Megophrys* toads here have been probably experienced complicated evolutionary history, for example, introgression between clades or incomplete lineage sorting and niche divergences in microhabitats. Anyway, it is urgent for us to explore the problems because these toads are suffering from increasing threats from human activities and climatic changes.

## Introduction

Species are the basic units of biodiversity, and species taxonomy is central to biodiversity explorations, further contributing to evolutionary biology, conservation biology and other categories of biological studies (Queiroz 2007; Condon et al. 2008; Wheeler et al. 2012). Increasing numbers of studies have advocated integrative taxonomy mainly because the findings from different disciplines would improve rigor (Dayrat 2005; Pante et al. 2014; Gómez Daglio and Dawson 2019). Integrative taxonomy has strongly promoted the discovery of cryptic species either in the understudied taxa (Larsen 2001; Bickford et al. 2007; Burns et al. 2008; Yoder et al. 2005) or in well-studied biomes (Rissler and Apodaca 2007; Stockman and Bond 2007). It could also resolve the taxonomic confusions like through demonstration of conspecificity of described species (Petrusek et al. 2008; Seifert 2009). And finally, multiple disciplines may further bring out clues for understanding the evolutionary processes of species for example in cases of disagreement among disciplines (DeSalle and Giddings 1986; Degnan and Rosenberg 2009; Thielsch et al. 2017).

The Asian horned toad *Megophryssensu lato* Kuhl and Van Hasselt, 1822 (Anura, Megophryidae Bonaparte, 1850) widely inhabit mountain forests in the tropical and subtropical regions of Asia, ranging from India to south-central China and south to the Sundas and the Philippines (Frost 2020). The generic classifications of the group have been controversial for a long time (e.g., Tian and Hu 1983; Dubois 1987; Rao and Yang 1997; Lathrop 1997; Jiang et al. 2003; Delorme et al. 2006; Fei et al. 2009; Fei and Ye 2016; Chen et al. 2016; Mahony et al. 2017; Liu et al. 2018; Frost 2020). Most recent phylogenetic studies, however, clustered all members of the group into a monophyletic group (Chen et al. 2016; Mahony et al. 2017; Liu et al. 2018; Li et al. 2018; Liu et al. 2020; Wang et al. 2020), which was defined as one big genus *Megophryssensu lato* by Mahony et al. (2017). The genus currently contains 95 species, of which, noticeably, 39 species were discovered in this decade (Frost 2020; Liu et al. 2020; Wang et al. 2020). What’s more, molecular phylogenetic studies still put forward dozens of cryptic species in the group (Chen et al. 2016; Liu et al. 2018). Misleading taxonomic judgements without precise and adequate comparisons and insufficient field work often hinder the discovery of cryptic diversity in the group (Mahony et al. 2018; Liu et al. 2018). Hence, comprehensive examinations with multiple data (e.g., molecular phylogenetic, morphological, and bioacoustics data) are needed for describing new taxon and furtherly recognizing underestimated species diversity in this diverse group.

Himalaya Mountains holds high level of biodiversity, and with increasingly deep surveys, species diversity in this region was indicated to be much underestimated. For example, just in Medog County, Tibet Autonomous Region, China in the eastern corner of Himalayas, several new frog or toad species has been found in recent years (e.g., Jiang et al. 2012; Jiang et al. 2016a, b, c). In Medog County, two *Megophrys* species has been recorded, i.e., *M. pachyproctus* Huang, 1981 and *M. medogensis* Fei, Ye and Huang 1983. Nevertheless, for ca. four decades, there have been only incomplete morphological reports (e.g., Fei et al. 2009; Fei and Ye 2016) or separate molecular data for them (Chen et al. 2016; Liu et al. 2018) but no detailed evaluation on taxonomic profiles of their populations especially using multiple disciplines. According to the hypothesis “lots of cryptic species in *Megophrys*” (Chen et al. 2016; Liu et al. 2018), it is expected that the toad populations in this high-profile biodiversity hotspot may contain cryptic species.

In recent years, we conducted a series of field surveys in Medog County, Tibet Autonomous Region, China, and collected a series of specimens of *Megophryssensu lato.* Based on molecular phylogenetic, morphological, osteological and bioacoustics data, we will explore the species composition of the Asian horned toad *Megophrys* in Medog County, Tibet Autonomous Region, China in the eastern corner of Himalayas. Our multiple-data comparisons proposed that the specimens contained two undescribed species. Herein we describe them as two new species.

## Materials and methods

### Sampling

**Figure 1.**
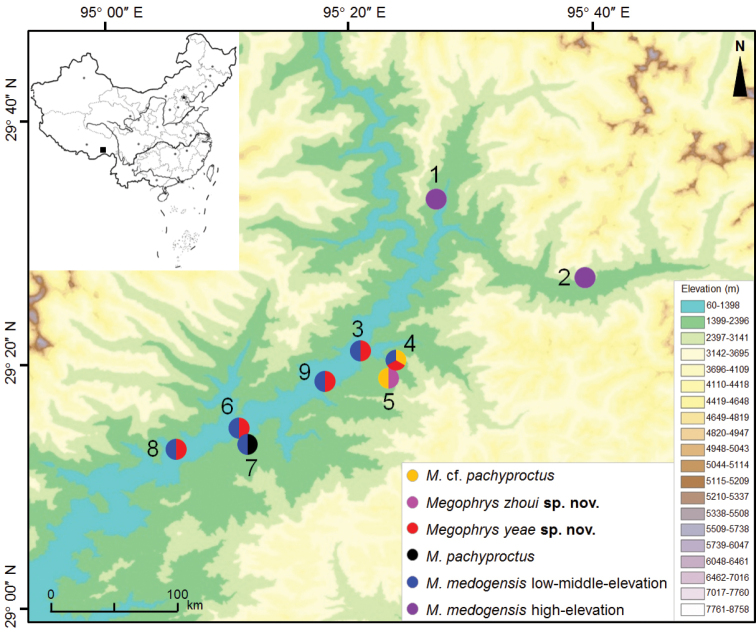
Distributional localities for specimens of the *Megophrys* species used in this study in Medog County, Tibet Autonomous Region, China. **1** 80k **2** Gedang village **3** vicinity of Medog urban area **4** Bari village **5** vicinity of Renqingbeng Temple **6** Beibeng village **7** Gelin village **8** Didong village **9** Yarang village. Species were denoted as different color.

A total of 50 *Megophrys* specimens was collected from nine sites in Medog County, Tibet Autonomous Region, China (Fig. 1; for voucher numbers see Table 1, Suppl. material 1: Tables S1, S2). The specimens were identified as four species, i.e., *M. medogensis*, M. cf. pachyproctus, and the two undescribed species (*Megophrys zhoui* sp. nov. and *Megophrys yeae* sp. nov.) based on morphology. Megophrys cf. pachyproctus was defined because the specimens were collected from the type locality of *M. pachyproctus* (a stream in Gelin village, Medog County), and they are morphologically similar to the holotype of *M. pachyproctus* although with some morphological differences. For caution, we regarded *M. pachyproctus* and M. cf. pachyproctus as two groups in the following analyses and descriptions. In addition, for comparison, we also divided *M. medogensis* specimens into two groups, i.e., high-elevation group (above ca. 2100 m a.s.l.) and low-middle-elevation group (500–1600 m). The high-elevation group contained five tadpoles collected from 80k and Gedang village, and the low-middle-elevation group contained five adult males, six adult females, and four tadpoles from the urban area of Medog town, Bari village, Beibeng village, Gelin village and Didong village (Fig. 1; Tables 1, Suppl. material 1: Tables S1, S2). Sex and maturity of each toad were determined by direct observation of advertisement calls or inspection of vocal sac openings and gonads. The tadpoles were identified based on their phylogenetic positions after representatives of the population with almost identical morphology were sequenced.

**Table 1.** Sampling information and GenBank accession numbers of samples used in the molecular analyses.

ID	Species	Voucher number	Locality	12S	16S	COI	RAG1	CXCR-4
1	*M. medogensis* low-middle elevation	CIB022017061502	Gelin, Medog, Tibet, China	/	MN963236	MN964296	MN984365	/
2		CIB022017060801	Beibeng, Medog, Tibet, China	/	MN963245	MN964287	MN984356	/
3		CIB022017060904	Didong, Medog, Tibet, China	/	MN963244	MN964288	MN984357	/
4		CIB022017061808	Bari, Medog, Tibet, China	/	MN963225	MN964306	MN984376	/
5		CIB022017061810	Bari, Medog, Tibet, China	/	MN963223	MN964308	MN984378	/
6		CIB022017061801	Bari, Medog, Tibet, China	/	MN963230	MN964301	MN984371	/
7		CIB022017061604	Beibeng, Medog, Tibet, China	/	MN963232	MN964299	MN984369	/
8		CIB022017061601	Beibeng, Medog, Tibet, China	/	MN963235	MN964297	MN984366	/
9		CIB022017061602	Beibeng, Medog, Tibet, China	/	MN963234	MN964298	MN984367	/
10		CIB022017061603	Beibeng, Medog, Tibet, China	/	MN963233	/	MN984368	/
11		CIB022017061501	Gelin, Medog, Tibet, China	/	MN963237	MN964295	MN984364	/
12		CIB022017061404DD	Didong, Medog, Tibet, China	/	MN963240	MN964292	MN984361	/
13		CIB022017061406MT	Suburb Medog, Tibet, China	/	MN963239	MN964293	MN984362	/
14		CIBMT1710104	Bari, Medog, Tibet, China	/	MN963212	MN964317	MN984385	/
15		CIBMT1710101	Yadong, vicinity of suburb Medog, Tibet, China	/	MN963213	MN964316	MN984384	/
16		KIZ06621	Beibeng, Tibet, China	/	KX811767	KX812082	KX812197	/
17	*M. medogensis* unknown elevation	SYSa002932	Motuo, Tibet, China	MH406458	MH406725	MH406177	MH404950	/
18	*M. glandulosa*	SYSa003795	Jingdong County, Yunnan, China	MH406493	MH406760	MH406219	MH404995	/
19	*M. medogensis* high elevation	CIB022017062002	80K, Medog, Tibet, China	/	MN963219	MN964310	/	/
20		CIB022017062003	80K, Medog, Tibet, China	/	MN963218	MN964311	/	/
21		CIBMT1710106	Gutang (Gedang), Medog, Tibet, China	/	MN963211	MN964318	MN984386	/
22		CIBMT1710107	Gutang (Gedang), Medog, Tibet, China	/	MN963210	MN964319	MN984387	/
23		CIBMT1710112	80K, Medog, Tibet, China	MN963176	MN963209	MN964320	MN984388	/
24	*M. medogensis* unknown elevation	SYSa002934	Motuo, Tibet, China	MH406459	MH406726	MH406178	MH404952	/
25	*M. mangshanensis*	KIZ021786	Nanling National Forest Park, Guangdong, China	/	KX811790	KX812079	KX812194	/
26	*M. maosonensis*	ROM 16679	Tam Dao, Vinh Phuc, Vietnam	/	KX811784	KX812081	KX812196	/
27	*M. periosa*	BNHS 6055 [SDBDU 2009.793]	28°12'33.96"N, 94°59'10.02"E	MH647522	MH647522	MH647529	MH647553	MH647537
28	*M. himalayana*	BNHS 6050 [SDBDU 2009.1227]	27°4'56.52"N, 92°34'50.22"E	MH647526	MH647526	/	MH647554	MH647538
29	*M. flavipunctata*	SDBDU 2009.297	East Khasi Hills dist, Meghalaya, India	KY022307	KY022307	MH647536	KY022352	KY022330
30	*M. robusta*	SDBDU 2011.1057	Darjeeling, West Bengal, India	KY022314	KY022314	/	KY022365	KY022343
31	*M. oreocrypta*	SDBDU 2009.1104	West Garo Hills dist, Meghalaya, India	KY022306	KY022306	/	KY022351	KY022329
32	*M. major*	SDBDU 2007.229	Kohima dist, Nagaland, India	MH647514	MH647514	/	MH647550	MH647540
33	*M. zhangi*	KIZ014278	Zhangmu, Tibet, China	/	KX811765	KX812084	KX812200	/
34	*M. monticola* middle elevation	SDBDU 2011.420	Darjeeling dist, West Bengal, India	MH647510	MH647510	/	KY022359	KY022337
35	*M. monticola* high elevation	SDBDU 2011.1047	Darjeeling dist, West Bengal, India	KY022312	KY022312	/	KY022358	KY022336
36		ZSI11401	Kabi, North district, Sikkim, India	/	KX894667	/	/	/
37	*M. lekaguli*	FMNH 265955	Pang Si Da, Sa Kaeo, Thailand	KY022214	KY022214	/	KY022241	KY022177
38	*M. auralensis*	NCSM 79599	Aural, Kampong Speu, Cambodia	/	KX811807	/	/	/
39	*M. takensis*	FMNH 261711	Khlong Lan National Park, Kampaeng, Thailand	KY022215	KY022215	/	KY022246	KY022183
40	M. cf. parva	KIZ048507	Tongbiguan Nature Reserve, Yunnan, China	/	KX811796	KX812071	KX812180	/
41	*M. zunhebotoensis*	RGK 0041	Nagaland, India	KY022322	KY022322	/	KY022367	KY022345
42	*M. serchhipii*	SDBDU 2009.612	Tripura, India	KY022323	KY022323	/	KY022366	KY022344
43	*M. ancrae*	SDBDU 2009.727	27°29.833'N 96°23.467'E	KY022318	KY022318	/	KY022350	KY022328
44	*M. oropedion*	SDBDU 2009.299	Mawphlang, Mawphlang Sacred Forest, East Khasi Hills, Meghalaya, India	KY022317	KY022317	/	KY022360	KY022338
45	*M. megacephala*	ZSI A 11213	East Khasi Hills, northern Meghalaya, India	KY022315	KY022315	/	KY022357	KY022335
46	*M. aceras*	LSUHC 7038	Tremengor Forest, Perak, Peninsular Malaysia, Malaysia	/	GQ995534	/	/	/
47	*M. longipes*	IABHU 21101	Genting highland, Malaysia	/	AB530656	/	/	/
48	*Megophrys yeae* sp. nov.	CIB201706MT01	Didong, Medog, Tibet, China	MN963172	MN963217	MN964312	MN984380	/
49		CIB201706MT02	Beibeng, Medog, Tibet, China	MN963173	MN963216	MN964313	MN984381	/
50		CIB201706MT03	Suburb of Medog, Tibet, China	MN963174	MN963215	MN964314	MN984382	/
51		CIB022017061102	Didong, Medog, Tibet, China	MN963162	MN963243	MN964289	MN984358	/
52		CIB022017061407b	Beibeng, Medog, Tibet, China	MN963165	MN963238	MN964294	MN984363	MN984402
53		CIB022017061804	Bari, Medog, Tibet, China	MN963167	MN963229	MN964302	MN984372	MN984403
54		CIB022017061809	Bari, Medog, Tibet, China	MN963171	MN963224	MN964307	MN984377	/
55		CIB022017061811	Bari, Medog, Tibet, China	/	MN963222	MN964309	MN984379	/
56		CIB022017061103	Didong, Medog, Tibet, China	MN963163	MN963242	MN964290	MN984359	/
57		CIB022017061104	Didong, Medog, Tibet, China	MN963164	MN963241	MN964291	MN984360	/
58		CIB022017061606	Beibeng, Medog, Tibet, China	MN963166	MN963231	MN964300	MN984370	/
59		CIBMT171064	Yadong, vicinity of suburb Medog, Tibet, China	MN963187	MN963198	/	MN984399	/
60		CIBMT171065	Yarang, Medog, Tibet, China	MN963188	MN963197	/	MN984400	/
61		CIBMT171066	Yarang, Medog, Tibet, China	MN963189	MN963196	/	MN984401	/
62		KIZ010978	Beibeng, Tibet, China	/	KX811908	KX812153	KX812265	/
63		KIZ011175	Beibeng, Tibet, China	/	KX811909	KX812154	KX812266	/
64	*M. vegrandis*	SDBDU 2009.1272 /ZSI A 11605	27°06.067'N 92°31.642'E	KY022305	KY022305	/	KY022349	KY022327
65	Megophrys cf. pachyproctus	CIBMT171053	Renqinbeng, Medog, Tibet, China	MN963178	MN963207	MN964322	MN984390	/
66		CIBMT171060	Renqinbeng, Medog, Tibet, China	MN963185	MN963200	MN964329	MN984397	/
67		CIBMT171062	Renqinbeng, Medog, Tibet, China	MN963186	MN963199	MN964330	MN984398	/
68		CIB022017061813	Bari, Medog, Tibet, China	/	MN963220	/	/	/
69		CIBMT171054	Renqinbeng, Medog, Tibet, China	MN963179	MN963206	MN964323	MN984391	/
70		CIBMT171052	Renqinbeng, Medog, Tibet, China	MN963177	MN963208	MN964321	MN984389	/
71		CIB201706MT04	Bari, Medog, Tibet, China	MN963175	MN963214	MN964315	MN984383	/
72		CIB022017061805	Bari, Medog, Tibet, China	MN963168	MN963228	MN964303	MN984373	MN984404
73		CIB022017061806	Bari, Medog, Tibet, China	MN963169	MN963227	MN964304	MN984374	MN984405
74		CIB022017061807	Bari, Medog, Tibet, China	MN963170	MN963226	MN964305	MN984375	MN984406
75		CIB022017061812	Bari, Medog, Tibet, China	/	MN963221	/	/	/
76	Megophrys cf. pachyproctus	CIBMT171055	Renqinbeng, Medog, Tibet, China	MN963180	MN963205	MN964324	MN984392	/
77		CIBMT171056	Renqinbeng, Medog, Tibet, China	MN963181	MN963204	MN964325	MN984393	/
78		CIBMT171057	Renqinbeng, Medog, Tibet, China	MN963182	MN963203	MN964326	MN984394	/
79		CIBMT171058	Renqinbeng, Medog, Tibet, China	MN963183	MN963202	MN964327	MN984395	/
80		CIBMT171059	Renqinbeng, Medog, Tibet, China	MN963184	MN963201	MN964328	MN984396	/
81	*M. xianjuensis*	CIBXJ190503	Xianju, Zhejiang, China	/	MN563758	MN563774	/	/
82	*M. lishuiensis*	WYF00169	Lishui, Zhejiang, China	/	KY021418	/	/	/
83	*M. shunhuangensis*	HNNU16SH02	Shunhuang Mountains, Hunan, China	MK836034	MK836037	/	/	/
84	*M. brachykolos*	SYSa002258	Hong Kong, China	MF667851	KJ560403	MH406120	MH404888	/
85	*M. kuatunensis*	SYSa003449	Guadun, Fujian, China	MF667850	MF667881	MH406206	MH404982	/
86	*M. dongguanensis*	SYSa001971/ CIB110006	Mt. Yinping, Dongguan City, Guangdong, China	/	MK524097	MK524128	/	/
87	*M. nankunensis*	SYSa004498	Mt. Nankun, Huizhou City,Guangdong, China	/	MK524108	MK524139	/	/
88	*M. wugongensis*	SYSa002610	Wugongshan Scenic Area, Anfu County, Jiangxi, China	/	MK524114	MK524145	/	/
89	*M. ombrophila*	NJFU2015201 KRM15	Mt. Wuyi, Fujian, China	KX856422	KX856401	/	/	/
90	*M. obesa*	SYSa002271	Heishiding, Guangdong, China	MH406410	KJ579121	MH406123	MH404891	/
91	*M. lini*	SYSa002381	Mt. Jinggang, Jiangxi, China	MF667842	MF667874	MH406135	MH404903	/
92	*M. nanlingensis*	SYSa001959	Nanling Nature Reserve, Shaoguan City, Guangdong, China	/	MK524111	MK524142	/	/
93	*M. cheni*	SYSa002126	Taoyuandong, Hunan, China	MH406389	MH406659	MH406096	MH404864	/
94	*M. insularis*	SYSa002169	Nan’ao Island, Guangdong, China	MH406393	MH406663	MH406103	MH404871	/
95	*M. jinggangensis*	SYSa004824	Mt. Sifang, Hunan, China	MH406590	MH406857	MH406319	MH405100	/
96	*M. caudoprocta*	SYSa004281	Zhangjiajie, Hunan, China	MH406528	MH406795	MH406257	MH405036	/
97	*M. tuberogranulatus*	SYSa004310	Zhangjiajie, Hunan, China	MH406534	MH406801	MH406263	MH405042	/
98	*M. wushanensis*	SYSa003008	Mt. Wu, Hubei, China	MH406465	MH406732	MH406184	MH404959	/
99	*M. leishanensis*	SYSa002213	Mt. Leigong, Guizhou, China	MH406403	MH406673	MH406113	MH404881	/
100	*M. acuta*	SYSa002276	Heishiding, Guangdong, China	MH406413	KJ579124	MH406126	MH404894	/
101	*M. boettgeri*	SYSa004149	Mt. Wuyi, Fujian, China	MF667847	MF667878	MH406247	MH405026	/
102	*M. huangshanensis*	SYSa002703	Huangshan, Anhui, China	MF667854	MF667883	MH406161	MH404929	/
103	*M. liboensis*	GNUG20150813001	Libo Country, Guizhou, China	MF285242	MF285253	/	/	/
104	*M. jiulianensis*	SYSa002107	Mt. Jiulian, Ganzhou City, Jiangxi, China	/	MK524099	MK524130	/	/
105	*M. mufumontana*	SYSa006390 CIB110012	Mt. Mufu, Pingjiang County, Hunan, China	/	MK524104	MK524135	/	/
106	*M. baolongensis*	KIZ019216	Baolong, Chongqing, China	/	KX811813	KX812093	KX812202	/
107	*M. sangzhiensis*	SYSa004306	Zhangjiajie, Hunan, China	MH406530	MH406797	MH406259	MH405038	/
108	*M. spinata*	SYSa002226	Mt. Leigong, Guizhou, China	MH406405	MH406675	MH406115	MH404883	/
109	*M. binlingensis*	SYSa005313	Wawu Shan, Sichuan, China	MH406625	MH406892	MH406354	MH405137	/
110	*M. wuliangshanensis*	SYSa003924	Mt. Wuliang, Yunnan, China	MH406504	MH406771	MH406230	MH405007	/
111	*M. jingdongensis*	SYSa003928	Mt. Wuliang, Yunnan, China	MH406506	MH406773	MH406232	MH405009	/
112	*M. daweimontis*	KIZ048997	Dawei Shan, Yunnan, China	/	KX811867	KX812125	KX812248	/
113	*M. omeimontis*	KIZ025765	Emei Shan, Sichuan, China	/	KX811884	KX812136	KX812223	/
114	*M. binchuanensis*	KIZ019441	Jizu Shan, Yunnan, China	/	KX811849	KX812112	KX812219	/
115	*M. rubrimera*	AMS R177676	Sa Pa, Lao Cai, Vietnam	/	MF536419	/	/	/
116	*M. jiangi*	CIBKKS20180722006	Kuankuosui Nature Reserve, Guizhou, China	/	MN107743	MN107748	/	/
117	*M. minor*	SYSa003209	Dujiangyan, Sichuan, China	MF667825	MF667862	MH406194	MH404969	/
118	*M. hansi*	AMCC 144729	Thua Tien Hue, A Luoi District, A Roang Commune, Viet Nam	KY022204	KY022204	/	KY022229	KY022165
119	*M. microstoma*	KU KUH 311601	Shiwan Dashang Nature Reserve, Guangxi, China	KY022200	KY022200	/	KY022234	KY022170
120	*M. gerti*	AMCC 106456	Quang Nam, Tra My Dist., Tra Don Commune, Viet Nam	KY022201	KY022201	/	KY022231	KY022167
121	*M. synoria*	FMNH 262778	Mondolkiri, Cambodia	KY022198	KY022198	/	KY022235	KY022171
122	*M. elfina*	ZMMU NAP-02658	Chu Pan Fan Mt, Chu Yang Sin N.P., Dak Lak Prov., Vietnam	KY425389	KY425389	/	/	/
123	*M. palpebralespinosa*	FMNH 258098	Phou Dendin National Biodiversity Conservation Area, Phongsaly, Laos	KY022209	KY022209	/	KY022238	KY022174
124	*M. intermedia*	FMNH 258093	Xe Kong, Kaleum District, Xe Sap National Biodiversity Conservation Area, Laos	KY022196	KY022196	/	KY022221	KY022157
125	*M. carinense*	CAS 243791	Khotama Camp, Yephyu, Dawei, Tanintharyi, Myanmar	KY022197	KY022197	/	KY022219	KY022155
126	*M. lancip*	MZB:Amp:22233	Ngarip, Ulubelu, Lampung, Sumatra, Indonesia	/	KY679891	/	/	/
127	*M. montana*	LSUMZ 81916	Sukabumi, Java, Indonesia	/	KX811927	KX812163	KX812281	/
128	*M. chuannanensis*	SYSa004926	Hejiang County, Sichuan, China	MH406635	MH406901	MH406364	MH405147	/
129	*M. feae*	SYSa003912	Jingdong County, Yunnan, China	MH406633	MH406899	MH406362	MH405145	MH450011
130	*M. popei*	SYSa001864	Taoyuandong, Hunan, China	MH406632	KM504256	MH406361	MH405144	/
131	*M. gigantica*	SYSa003883	Ailao Shan, Yunnan, China	MH406499	MH406766	MH406225	MH405001	MH450010
132	*M. wawuensis*	SYSa005311	Wawu Shan, Sichuan, China	MH406624	MH406891	MH406353	MH405136	/
133	*M. nankiangensis*	CIB ZYC517	Nanjiang, Sichuan, China	/	KX811900	/	/	/
134	*M. shapingensis*	KIZ014512	Liziping Nature Reserve, Sichuan, China	/	KX811904	KX812060	KX812274	/
135	*M. dringi*	UNIMAS 8948	Gunung Mulu, Sarawak, Malaysia	/	KJ831316	/	/	/
136	*M. nasuta*	MBH 5357	Bengkulu, Sumatra, Indonesia	KY022185	KY022185	/	KY022225	KY022161
137	*M. kalimantanensis*	FMNH 236525	Crocker Range National Park, Tenom Dist, Sabah, Borneo, Malaysia	DQ283342	DQ283342	/	/	/
138	*M. kobayashii*	UNIMAS 8148	Gunung Kinabalu National Park, Sabah, Malaysia	/	KJ831313	/	/	/
139	*M. baluensis*	voucher not preserved	Kinabalu, Borneo	DQ642146	DQ642121	/	/	/
140	*M. stejnegeri*	KU 314303	Pasonanca Natural Park, Zamboanga City, Philippines	/	KX811922	KX812052	KX812172	/
141	*M. edwardinae*	FMNH 273694	Bintulu, Sarawak, Malaysia	/	KX811918	KX812050	KX812168	/
142	*M. ligaya*	ZMMU NAP-05015	Palawan, Philippines	/	KX811919	KX812051	KX812169	/
143	*Leptolalax alpinus*	SYSa003927	Jingdong County, Yunnan, China	MH406639	MH406905	MH406368	MH405151	/
144	Leptobrachium cf. rakhinensis	SDBDU 2009.49	Trishna Wildlife Sanctuary, South Dist, Tripura state, India	KY022304	KY022304	/	KY022347	KY022325

In the field, after taking photographs, the toads and tadpoles were euthanized using isoflurane, and then the specimens were fixed in 75% ethanol. Tissue samples were taken and preserved separately in 95% ethanol prior to fixation. Specimens collected in this work were deposited in Chengdu Institute of Biology, Chinese Academy of Sciences (CIB, CAS). The Animal Care and Use Committee of Chengdu Institute of Biology, CAS provided full approval for this research (Number: CIB2016012301). Field work was approved by the Management Office of the Administration of Yarlung Zangbo Grand Canyon National Nature Reserve (YLZB000342).

### Molecular phylogenetic analyses

Total genomic DNA was extracted from each specimen collected in this study using QIAamp DNA Mini Kit (QIAGEN, Hilden, Germany), following manufacturer instructions. Three mitochondrial genes (12S rRNA, 16S rRNA, and COI) and two nuclear protein-coding genes (RAG1 and CXCR-4) were amplified and sequenced. Primer sequences were retrieved from literatures for 12S (Sumida et al. 2000), 16S (Simon et al. 1994), COI (Che et al. 2011), RAG1 (Mauro et al. 2004; Fu et al. 2007), and CXCR-4 (Biju and Bossuyt 2003) genes. PCR amplifications for mitochondrial genes were performed in a 30 μl volume reaction with the following conditions: an initial denaturing step at 95 °C for 4 min; 36 cycles of denaturing at 95 °C for 40 s, annealing at 55 °C (for 12S and 16S)/52 °C (for COI) for 40 s and extending at 72 °C for 70 s, and a final extending step of 72 °C for 10 min. Amplifications of nuclear genes were according to Mahony et al. (2017). PCR products were sequenced with both forward and reverse primers same as used in PCR. Sequencing was conducted using an ABI3730 automated DNA sequencer in Sangon Biotechnologies Co., Ltd. (Shanghai, China). New sequences were uploaded to GenBank (see Table 1).

For phylogenetic comparisons, corresponding sequences of *Megophrys* species were downloaded from GenBank especially for their holotypes and/or topotypes for which comparable sequences were available (Table 1). Corresponding sequences of one *Leptobrachium rakhinensis* and one *Leptobrachella khasiorum* (Table 1) were also downloaded and used as outgroups according to previous studies (Mahony et al. 2017; Chen et al. 2016).

Sequences were assembled and aligned using BioEdit v. 7.0.9.0 (Hall 1999) with default settings, and were further revised manually if necessary. To avoid bias in alignments, GBLOCKS v. 0.91.b (Castresana 2000) with default settings was used to extract regions of defined sequence conservation from the length-variable 12S and 16S fragments. The protein-coding gene (COI, RAG1, and CXCR-4) sequences were translated to amino acid sequences in MEGA v. 7.0 (Kumar et al. 2016), adjusted for open reading frames, and checked to ensure absence of premature stop codons. No-sequenced fragments were treated as missing data. At last, for phylogenetic analyses, two datasets were obtained, i.e., three-mitochondrial genes concatenated dataset of 12S+16S+COI and two-nuclear genes concatenated dataset of RAG1+CXCR-4.

Phylogenetic analyses were conducted on each dataset using maximum likelihood (ML) and Bayesian Inference (BI) methods, implemented in PhyML v. 3.0 (Guindon et al. 2010) and MrBayes v. 3.2 (Ronquist et al. 2012), respectively. For the phylogenetic analyses, each gene was regarded as one partition, and the best evolutionary model for each partition were chosen under the Bayesian Inference Criteria (BIC) using jModelTest v. 2.1.3 (Darriba 2012). The analyses selected GTR + I + G model for each mitochondrial gene, and HKY + I for each nuclear gene. For the ML tree, branch supports were drawn from 10000 non-parametric bootstrap replicates. In BI analyses, the parameters for each partition were unlinked, and branch lengths were allowed to vary proportionately across partitions. Two runs each with four Markov chains were simultaneously run for 80 million generations with sampling every 1000 generations. The first 25% of trees were removed as the “burn-in” stage followed by calculations of Bayesian posterior probabilities at stationarity, and the 50% majority-rule consensus of the post burn-in trees sampled. Finally, genetic distance between species with uncorrected *p*-distance model on the 16S gene was estimated using MEGA.

### Morphological analyses

In total, 38 adult specimens of four species (the two undescribed species, *M. medogensis*, and M. cf. pachyproctus) were measured (Suppl. material 1: Table S1). The terminology and methods followed Mahony (2011). Measurements were taken with a dial caliper to the nearest 0.1 mm. Twenty-two characters of adult specimens were measured:

**EL** eye length (horizontal distance between the anterior and posterior borders of orbit);

**EN** eye-nostril length (distance from front of eye to the center of nostril);

**FAL** forearm length (distance from elbow to wrist);

**FIIIW** finger III width (largest width of tip of finger III);

**FIVW** finger IV width (largest width of tip of finger IV);

**FOL** foot length (distance from the proximal end of the inner metatarsal tubercle to the tip of the fourth digit);

**HAL** hand length (distance from wrist to tip of third digit);

**HL** head length (distance from the rear of the mandible to the tip of the snout);

**HLL** hindlimb length;

**HW** head width (distance between the posterior angles of jaw);

**IBE** internal back of eyes (the shortest distance between the posterior borders of the orbits);

**IFE** internal front of eyes (shortest distance between the anterior borders of orbits);

**IMT** ength of the inner metatarsal tubercle;

**IN** internarial distance (shortest distance between two nostrils);

**IUE** inter upper eyelid width (shortest distance between upper eyelids);

**SHL** shank length (distance from knee to ankle);

**SL** snout length (distance from tip of snout to anterior border of the orbit);

**SN** nostril-snout length (distance from center of the nostril to tip of the snout);

**SVL** snout-vent length (distance from the tip of the snout to the posterior edge of the vent);

**TFOL** tarsal-foot length (distance from heel to the tip of the fourth digit);

**TL** thigh length (distance from cloaca to knee);

**TYD** largest tympanum diameter;

**TYE** tympanum-eye distance (distance from the anterior border of the tympanum to the posterior orbital border);

**UEW** maximum upper eyelid width.

Thirteen tadpoles of four groups (i.e., *Megophrys yeae* sp. nov., M. cf. pachyproctus, and two elevation groups of *M. medogensis*) were measured (Suppl. material 1: Table S2). The stages of tadpoles were identified following Gosner (1960). Seventeen morphometric characters of tadpoles were measured:

**BH** maximum body height;

**BL** body length (distance from tip of snout to trunk-tail junction);

**BW** maximum body width;

**ED** maximum eye diameter;

**IND** internasal distance (distance between center of two naris);

**LF** maximum height of lower tail fin;

**NE** naris-eye distance (distance from center of naris to anterior corner of eye);

**ODW** oral disc width (largest width of oral disc);

**PP** interpupilar distance;

**RN** rostro-narial distance (distance from tip of snout to center of naris);

**SS** snout-spiracle distance (distance from tip of snout to opening of spiracle);

**SU** snout-upper fin distance (distance from snout to beginning of upper tail fin);

**TAL** tail length (distance between posterior side of opening of cloaca to tip of tail);

**TMH** maximum tail muscle width;

**TMW** maximum tail muscle height;

**TOL** total length;

**UF** maximum height of upper tail fin.

For morphometric comparisons, the corresponding morphometric data of the holotype and two topotypes of *M. vegrandis* were retrieved from Mahony et al. (2013), and that of the allotype and one paratype of *M. pachyproctus* from Huang and Fei (1981). To reduce the impact of allometry, the correct value from the ratio of each measurement to SVL was calculated and then log-transformed for the following morphometric analyses. Mann-Whitney *U* test was used to test the significance of difference on each character between different species in each gender group. In the analyses for male group, 13 characters of 28 individuals of five species (*Megophrys yeae* sp. nov., M. cf. pachyproctus, *M. pachyproctus*, *M. medogensis*, and *M. vegrandis*) were included, and for female, 26 characters of 13 individuals of four species (*Megophrys yeae* sp. nov., *Megophrys zhoui* sp. nov., M. cf. pachyproctus, and *M. medogensis*) were included. The significance level was set at 0.05. The analyses were carried out in R (R Development Core Team 2008).

The two undescribed species were compared with each other as well with other congeners of *Megophryssensu lato* on morphology. Comparative morphological data were obtained from literatures (Table 2). In addition, the holotype of *M. pachyproctus* and topotypes of *M. medogensis* were also examined for comparisons (Suppl. material 1: Tables S1, S2).

**Table 2.** References utilized for morphological characters of congeners of the genus *Megophrys*.

No.	Species	Literature obtained
1	*Megophrys aceras* Boulenger, 1903	Bourret 1942; Munir et al. 2018
2	*Megophrys acuta* Wang, Li, and Jin, 2014	Li et al. 2014
3	*Megophrys ancrae* Mahony, Teeling, and Biju, 2013	Mahony et al. 2013
4	*Megophrys angka* Wu, Suwannapoom, Poyarkov, Chen, Pawangkhanant, Xu, Jin, Murphy, and Che, 2019	Wu et al. 2019
5	*Megophrys auralensis* Ohler, Swan, and Daltry, 2002	Ohler et al. 2002
6	*Megophrys baluensis* Boulenger, 1899	Boulenger 1899, 1908
7	*Megophrys baolongensis* Ye, Fei, and Xie, 2007	Ye et al. 2007; Fei and Ye 2016
8	*Megophrys binchuanensis* Ye and Fei, 1995	Ye et al. 1995; Fei and Ye 2016
9	*Megophrys binlingensis* Jiang, Fei, and Ye, 2009	Fei et al. 2009
10	*Megophrys boettgeri* Boulenger, 1899	Boulenger 1908; Fei et al. 2009
11	*Megophrys brachykolos* Inger and Romer, 1961	Inger et al. 1961; Fei et al. 2009; Li et al. 2014
12	*Megophrys carinense* Boulenger, 1889	Boulenger 1908; Bourret 1942
13	*Megophrys caudoprocta* Shen, 1994	Shen 1994; Shen et al. 2013
14	*Megophrys cheni* Wang and Liu, 2014	Wang et al. 2014
15	*Megophrys chuannanensis* Fei, Ye, and Huang, 2001	Fei and Ye 2001; Fei et al. 2009
16	*Megophrys damrei* Mahony, 2011	Mahony 2011
17	*Megophrys daweimontis* Rao and Yang, 1997	Rao and Yang 1997; Fei and Ye 2016
18	*Megophrys dongguanensis* Wang and Wang, 2019	Wang et al. 2019
19	*Megophrys dringi* Inger, Stuebing, and Tan, 1995	Inger et al. 1995; Oberhummer et al. 2014
20	*Megophrys edwardinae* Inger, 1989	Inger et al. 1989
21	*Megophrys elfina* Poyarkov, Duong, Orlov, Gogoleva, Vassilieva, Nguyen, Nguyen, Nguyen, Che, and Mahony, 2017	Poyarkov et al. 2017
22	*Megophrys fansipanensis* Tapley, Cutajar, Mahony, Nguyen, Dau, Luong, Le, Nguyen, Nguyen, Portway, Luong, and Rowley, 2018	Tapley et al. 2018
23	*Megophrys feae* Boulenger, 1887	Boulenger 1908; Fei et al. 2009
24	*Megophrys feii* Yang, Wang, and Wang, 2018	Yang et al. 2018
25	*Megophrys flavipunctata* Mahony, Kamei, Teeling, and Biju, 2018	Mahony et al. 2018
26	*Megophrys gerti* Ohler, 2003	Poyarkov et al. 2017
27	*Megophrys gigantica* Liu, Hu, and Yang, 1960	Liu et al. 1960; Fei et al. 2009
28	*Megophrys glandulosa* Fei, Ye, and Huang, 1990	Fei et al. 1990; Fei et al. 2009; Fei and Ye 2016
29	*Megophrys hansi* Ohler, 2003	Ohler 2003
30	*Megophrys himalayana* Mahony, Kamei, Teeling, and Biju, 2018	Mahony et al. 2018
31	*Megophrys hoanglienensis* Tapley, Cutajar, Mahony, Nguyen, Dau, Luong, Le, Nguyen, Nguyen, Portway, Luong, and Rowley, 2018	Tapley et al. 2018
32	*Megophrys huangshanensis* Fei and Ye, 2005	Fei and Ye 2005, 2016; Fei et al. 2009
33	*Megophrys insularis* Wang, Liu, Lyu, Zeng, and Wang, 2017	Wang et al. 2017a
34	*Megophrys intermedia* Smith, 1921	Smith 1921
35	*Megophrys Jiangi* Liu, Li, Wei, Xu, Cheng, Wang and Wu, 2020	Liu et al. 2020
36	*Megophrys jingdongensis* Fei and Ye, 1983	Fei at al. 1983, 2009; Fei and Ye 2016
37	*Megophrys jinggangensis* Wang, 2012	Wang et al. 2012
38	*Megophrys jiulianensis* Wang, Zeng, Lyu, and Wang, 2019	Wang et al. 2019
39	*Megophrys kalimantanensis* Munir, Hamidy, Matsui, Iskandar, Sidik, and Shimada, 2019	Munir et al. 2019
40	*Megophrys kobayashii* Malkmus and Matsui, 1997	Malkmus and Matsui 1997
41	*Megophrys koui* Mahony, Foley, Biju, and Teeling, 2017	Yang 1991
42	*Megophrys kuatunensis* Pope, 1929	Pope 1929; Fei et al. 2009; Tapley et al. 2017
43	*Megophrys lancip* Munir, Hamidy, Farajallah, and Smith, 2018	Munir et al. 2018
44	*Megophrys leishanensis* Li, Xu, Liu, Jiang, Wei, and Wang, 2019 “2018”	Li et al. 2018a
45	*Megophrys lekaguli* Stuart, Chuaynkern, Chan-ard, and Inger, 2006	Stuart et al. 2006a
46	*Megophrys liboensis* Zhang, Li, Xiao, Li, Pan, Wang, Zhang, and Zhou, 2017	Zhang et al. 2017
47	*Megophrys ligayae* Taylor, 1920	Taylor 1920
48	*Megophrys lini* Wang and Yang, 2014	Wang et al. 2014
49	*Megophrys lishuiensis* Wang, Liu and Jiang, 2017	Wang et al. 2017b
50	*Megophrys longipes* Boulenger, 1886	Boulenger 1908; Bourret 1942
51	*Megophrys major* Boulenger, 1908	Boulenger 1908
52	*Megophrys mangshanensis* Fei and Ye, 1990	Fei et al. 1990; Fei and Ye 2016
53	*Megophrys maosonensis* Bourret, 1937	Bourret 1942
54	*Megophrys medogensis* Fei, Ye, and Huang, 1983	Fei at al. 1983, 2009; Fei and Ye 2016; This paper
55	*Megophrys megacephala* Mahony, Sengupta, Kamei, and Biju, 2011	Mahony et al. 2011
56	*Megophrys microstoma* Boulenger, 1903	Fei et al. 2009
57	*Megophrys minor* Stejneger, 1926	Stejneger 1926; Li et al. 2014; Fei and Ye 2016
58	*Megophrys montana* Kuhl and Van Hasselt, 1822	Munir et al. 2018
59	*Megophrys monticola* Günther, 1864	Mahony et al. 2018
60	*Megophrys mufumontana* J. Wang, Lyu, and Y.Y. Wang, 2019	Wang et al. 2019
61	*Megophrys nankiangensis* Liu and Hu, 1966	Fei et al. 2009
62	*Megophrys nankunensis* Wang, Zeng, and. Wang, 2019	Wang et al. 2019
63	*Megophrys nanlingensis* Lyu, J. Wang, Liu, and Y.Y. Wang, 2019	Wang et al. 2019
64	*Megophrys nasuta* Schlegel, 1858	Mahony et al. 2018
65	*Megophrys obesa* Wang, Li, and Zhao, 2014	Li et al. 2014
66	*Megophrys ombrophila* Messenger and Dahn, 2019	Messenger et al. 2019
67	*Megophrys omeimontis* Liu, 1950	Fei et al. 2009; Fei and Ye 2016
68	*Megophrys oreocrypta* Mahony, Kamei, Teeling, and Biju, 2018	Mahony et al. 2018
69	*Megophrys oropedion* Mahony, Teeling, and Biju, 2013	Mahony et al. 2013
70	*Megophrys pachyproctus* Huang, 1981	[Bibr B43][Bibr B42]; This paper
71	*Megophrys palpebralespinosa* Bourret, 1937	Bourret 1942
72	*Megophrys parallela* Inger and Iskandar, 2005	Inger and Iskandar 2005
73	*Megophrys parva* Boulenger, 1893	Boulenger 1908; Deuti et al. 2017
74	*Megophrys periosa* Mahony, Kamei, Teeling, and Biju, 2018	Mahony et al. 2018
75	*Megophrys popei* Zhao, Yang, Chen, Chen, and Wang, 2014	Zhao et al. 2014
76	*Megophrys robusta* Boulenger, 1908	Boulenger 1908; Mahony et al. 2018
77	*Megophrys rubrimera* Tapley, Cutajar, Mahony, Chung, Dau, Nguyen, Luong, and Rowley, 2017	Tapley et al. 2017
78	*Megophrys sangzhiensis* Jiang, Ye, and Fei, 2008	Jiang et al. 2008
79	*Megophrys serchhipii* Mathew and Sen, 2007	Mathew and Sen 2007
80	*Megophrys shapingensis* Liu, 1950	Liu et al. 1950; Fei et al. 2009
81	*Megophrys shuichengensis* Tian and Sun, 1995	Tian and Sun 1995; Fei et al. 2009
82	*Megophrys shunhuangensis* Wang, Deng, Liu, Wu, and Liu, 2019	Wang et al. 2019a
83	*Megophrys spinata* Liu and Hu, 1973	Fei et al. 2009; Fei and Ye 2016
84	*Megophrys stejnegeri* Taylor, 1920	Taylor 1920
85	*Megophrys synoria* Stuart, Sok, and Neang, 2006	Stuart et al. 2006b
86	*Megophrys takensis* Mahony, 2011	Mahony 2011
87	*Megophrys tuberogranulata* Shen, Mo and Li, 2010	Mo et al. 2010
88	*Megophrys vegrandis* Mahony, Teeling, Biju, 2013	Mahony et al. 2013
89	*Megophrys wawuensis* Fei, Jiang, and Zheng, 2001	Fei et al. 2009
90	*Megophrys wugongensis* J. Wang, Lyu, and Y.Y. Wang, 2019	Wang et al. 2019
91	*Megophrys wuliangshanensis* Ye and Fei, 1995	Fei et al. 2009; Fei and Ye 2016
92	*Megophrys wushanensis* Ye and Fei, 1995	Fei et al. 2009; Fei and Ye 2016
93	*Megophrys xianjuensis* Wang, Wu, Peng, Shi, Lu and Wu, 2020	Wang et al. 2020
94	*Megophrys zhangi* Ye and Fei, 1992	Ye and Fei 1992; Fei et al. 2009; Fei and Ye 2016
95	*Megophrys zunhebotoensis* Mathew and Sen, 2007	Mathew and Sen 2007

### Bioacoustics

We recorded advertisement calls of three species: six males (CIB022017061804, CIB022017061101–CIB022017061103, CIBMT171064, and one unvouchered individual) of *Megophrys yeae* sp. nov., three males (CIB022017061805–CIB022017061807) of M. cf. pachyproctus, and three unvouchered males of *M. medogensis* (Suppl. material 1: Table S3). Each calling individual was recorded at a distance between 0.5–1.0 m using a Philip VTR6900 digital voice recorder with a build-in microphone with sampling rate 96 kHz. Temperature was recorded using HTC-1 hygrothermograph. All callings were recorded between a relatively concentrated temperature range of 17–25 °C. Calls were analyzed using Raven Pro^©^ v.1.5 beta software (http://www.birds.cornell.edu/raven) with fast-Fourier transform (FFT) of 512 points, 50% overlap, and 188 Hz grid-spacing using Hanning windows. Sonograms and spectrograms were presented in figures using Praat (Boersma 2001) after de-noised using Audition 3. Terminology of advertisement call analyses and description followed Köhler et al. (2017). Call duration (ms), intercall interval (ms), number of calls per call group, call repetition rate (calls/s), number of pulses per call, and dominant frequency (kHz) were applied in measurement. To compare acoustic characteristics between the species, one-way ANOVA was conducted with LSD post hoc.

**Skull scanning.** The holotype CIB201706MT02 of *Megophrys yeae* sp. nov., holotype CIBMT171053 of *Megophrys zhoui* sp. nov., and the adult male CIB022017061805 of M. cf. pachyproctus were scanned. For comparisons, the holotype NWIPB 770650 of *M. pachyproctus* and the adult male topotype CIB022017061406 of *M. medogensis* were also scanned. In the high-resolution X-ray scanner (Quantum GX micro-CT Imaging System, PerkinElmer®), the specimens were scanned along the coronal axis at an image resolution of 1024× 1024 pixels. Segmentation and three-dimensional reconstruction of the CT images were made using VG57 Studio Max 2.2 (Volume Graphics, Heidelberg, Germany). Terminology of skull description follows Fei and Yei (2016).

## Results

### Phylogenetic analyses

**Figure 2.**
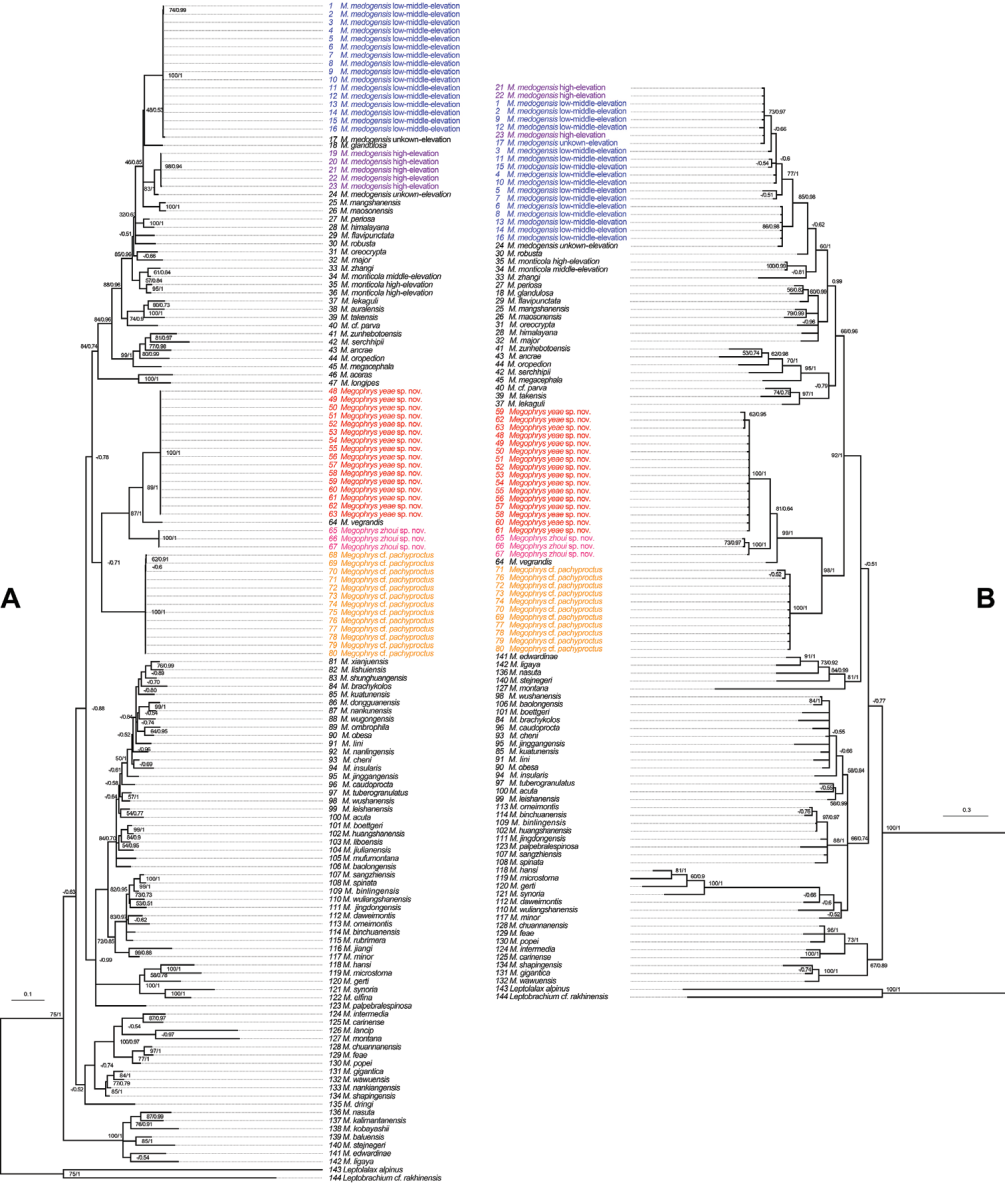
Phylogenetic trees respectively based on the mitochondrial DNA and nuclear DNA. **A** Maximum Likelihood (ML) tree based on the mitochondrial DNA **B**ML tree based on the nuclear DNA. ML bootstrap support/Bayesian posterior probability was denoted beside node. Samples 1–144 refer to Suppl. material 1: Table S1.

Aligned sequence matrix of mitochondrial DNA and nuclear DNA contained 2890 bp and 2058 bp, respectively. ML and BI analyses based on the mitochondrial DNA matrix resulted in essentially consistent topologies (Fig. 2A), and all analyses on nuclear DNA matrix also obtained generally consistent topologies (Fig. 2B), though some relationships were not r|solved in these trees.

All samples of *Megophryssensu lato* were strongly clustered into a clade in all trees. In all trees, each of the two new species was well supported as an independent clade, and all of them were then clustered into a big clade also containing M. cf. pachyproctus and *M. vegrandis*. In all trees, in this clade, M. cf. pachyproctus was indicated to be at the basal position. In mitochondrial DNA trees, the relationships of other three species were supported as (*Megophrys zhoui* sp. nov. (*M. vegrandis*, *Megophrys yeae* sp. nov.)), but in nuclear DNA trees, as (*M. vegrandis* (*Megophrys yeae* sp. nov., *Megophrys zhoui* sp. nov.)). This clade with the four species was phylogenetically far from the clade containing all samples of *M. medogensis* in all trees. As note, in nuclear DNA trees, *M. medogensis* was resolved as a monophyletic group because the high-elevation and low-middle-elevation groups of *M. medogensis* were nested into one clade, but in mitochondrial DNA trees, the low-middle-elevation group of *M. medogensis* was clustered as a clade sister to *M. robusta*, being paraphyly with the clade in comprising of the high-elevation group of *M. medogensis*.

Genetic distance among samples of each new species is below 0.4%, much lower than the interspecific distance of *Megophrys* (mean: 10.5%; range: 0.8%–26.1%; Suppl. material 1: Table S4). Genetic distance between *Megophrys zhoui* sp. nov. and other congeners was at least 4.0% (*Megophrys zhoui* sp. nov. vs. *M. vegrandis*), and that between *Megophrys yeae* sp. nov. and other congeners was at least 5.4% (*Megophrys yeae* sp. nov. vs. *M. vegrandis*). As note, genetic distance between the low-middle-elevation and high-elevation groups of *M. medogensis* was 5.0% on 16S gene. These values were much higher than interspecific genetic distance between many pairs of *Megophrys* species (Suppl. material 1: Table S4).

### Morphological analyses

On many morphometric characters, the two new species were significantly different from each other as well from *M. vegrandis*, *M. medogensis*, and *M. pachyproctus* (Table 3). In male, ten characters were significantly different at least between one pair of species, i.e., SVL, HL, SL, EL, UEW, TYD, FAL, HAL, SHL, and FOL (all P-values < 0.05; Table 3); and in female, 14 characters were significant different at least between one pair of species, i.e., SVL, HW, HL, SL, IFE, IBE, TYD, TYE, FAL, HAL, FIL, FIIIL, FIIIW, and FIVW (all P-values < 0.05; Table 3).

**Table 3.** Morphometric comparisons between the *Megophrys* species from the eastern corner of Himalayas. P-value is resulted from Mann-Whitney *U* test on each character between species. Significant level at 0.05 (* P-value < 0.05). Abbreviation for species name: MCP, M. cf. pachyproctus; MZ, *Megophrys zhoui* sp. nov.; MY, *Megophrys yeae* sp. nov.; MP, *M. pachyproctus*; MM, *M. medogensis*; and MV, *M. vegrandis*. See abbreviations for the morphological characters in Materials and methods section.

Sex	Character	MCP vs. MY	MCP vs. MZ	MCP vs. MM	MCP vs. MP	MCP vs. MV	MZ vs. MY	MZ vs. MM	MY vs. MP	MY vs. MM	MP vs. MV	MM vs. MV
**Female**	**SVL**	0.133	0.133	0.016*	/	/	0.333	0.095	/	0.095	/	/
	**HW**	0.133	0.267	0.016*	/	/	0.333	0.095	/	0.095	/	/
	**HL**	1.000	0.267	0.032*	/	/	1.000	0.571	/	0.190	/	/
	**SL**	0.533	0.267	0.032*	/	/	0.333	0.095	/	1.000	/	/
	**SN**	1.000	0.267	1.000	/	/	0.333	0.190	/	0.857	/	/
	**EN**	0.800	1.000	0.286	/	/	1.000	0.570	/	1.000	/	/
	**IN**	1.000	0.267	0.286	/	/	1.000	1.000	/	0.857	/	/
	**EL**	0.133	1.000	1.000	/	/	0.667	1.000	/	0.095	/	/
	**IUE**	0.533	0.533	0.111	/	/	1.000	1.000	/	1.000	/	/
	**UEW**	0.533	0.533	0.730	/	/	1.000	1.000	/	1.000	/	/
	**IFE**	0.800	0.267	0.111	/	/	1.000	0.950	/	0.571	/	/
	**IBE**	0.133	0.133	1.000	/	/	0.667	0.190	/	0.190	/	/
	**TYD**	0.533	0.133	0.032*	/	/	0.333	0.095	/	0.095	/	/
	**TYE**	0.800	0.133	0.016*	/	/	0.667	0.095	/	0.095	/	/
	**FAL**	0.133	0.800	0.286	/	/	0.333	0.381	/	0.095	/	/
	**HAL**	0.133	1.000	0.016*	/	/	0.333	0.095	/	0.095	/	/
	**FIL**	1.000	0.133	0.016*	/	/	0.333	0.095	/	0.095	/	/
	**FIIL**	0.133	0.533	0.016*	/	/	0.333	0.095	/	0.381	/	/
	**FIIIL**	0.133	0.267	0.286	/	/	0.333	1.000	/	0.095	/	/
	**FIVL**	1.000	0.533	0.730	/	/	0.333	0.381	/	0.381	/	/
	**TL**	0.133	0.533	0.413	/	/	0.333	0.571	/	1.000	/	/
	**SHL**	0.533	0.800	0.730	/	/	0.333	0.857	/	0.857	/	/
	**TFOL**	1.000	0.133	0.730	/	/	0.333	0.857	/	1.000	/	/
	**FOL**	1.000	0.267	1.000	/	/	0.333	0.571	/	1.000	/	/
	**FIIIW**	0.133	0.133	0.556	/	/	0.333	0.095	/	0.095	/	/
	**FIVW**	0.133	0.133	0.730	/	/	0.333	0.095	/	0.095	/	/
**Male**	**SVL**	0.001*	/	0.476	0.533	0.029*	/	/	/	0.005*	0.643	0.038*
	**HW**	0.446	/	1.000	0.533	0.486	/	/	/	0.180	0.286	0.067
	**HL**	0.599	/	0.038*	1.000	0.057	/	/	/	0.005*	0.143	0.171
	**SL**	0.521	/	0.610	0.533	0.200	/	/	/	0.125	0.286	0.067
	**IN**	0.262	/	0.257	1.000	0.686	/	/	/	1.000	1.000	0.352
	**EL**	0.262	/	0.380	0.533	0.886	/	/	/	0.000*	0.710	0.670
	**UEW**	0.133	/	0.190	1.000	0.029*	/	/	/	0.180	0.710	0.010*
	**TYD**	0.262	/	0.380	0.533	0.343	/	/	/	0.018*	1.000	0.010*
	**FAL**	0.002*	/	0.010*	1.000	0.029*	/	/	/	0.000*	0.710	0.010*
	**HAL**	0.133	/	0.010*	0.800	0.029*	/	/	/	0.000*	0.710	0.010*
	**SHL**	0.684	/	0.010*	0.533	0.343	/	/	/	0.102	0.710	0.914
	**TFOL**	0.212	/	0.171	1.000	0.343	/	/	/	0.964	0.643	0.171
	**FOL**	0.020*	/	1.000	0.533	0.886	/	/	/	0.007*	1.000	0.762

**Figure 3.**
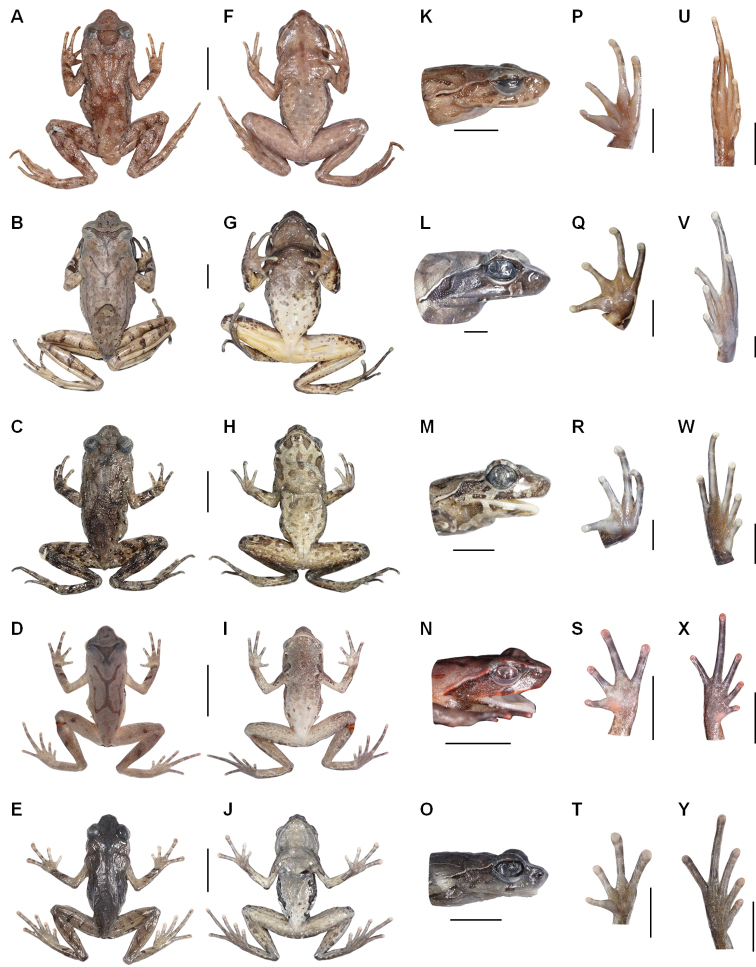
Photos of specimens of *Megophrys* species in Medog. **A–E** dorsal views of adult male holotype NWIPB770650 of *M. pachyproctus*, adult male topotype CIB022017061406 of *M. medogensis*, adult male CIB022017061805 of M. cf. pachyproctus, adult male holotype CIBMT171053 of *Megophrys zhoui* sp. nov., and adult male holotype CIB201706MT02 of *Megophrys yeae* sp. nov., respectively **F–J** ventral views of the specimens, respectively **K–O** lateral view of head of the specimens, respectively **P–T** ventral view of hand of the specimens, respectively **U–Y** ventral view of foot of the specimens, respectively. Scale bar for body view equal to 10 mm, and for partial view 5 mm.

On morphology, the two new species could be identified from each other as well as from their congeners by a series of characters (for morphological differences between the five groups of *Megophrys* species from Medog County see Suppl. material 1: Table S5; Fig. 3). Detailed comparisons on morphological characters between the new species and other congeners were demonstrated in detail in the sections for describing the new species.

**Figure 4.**
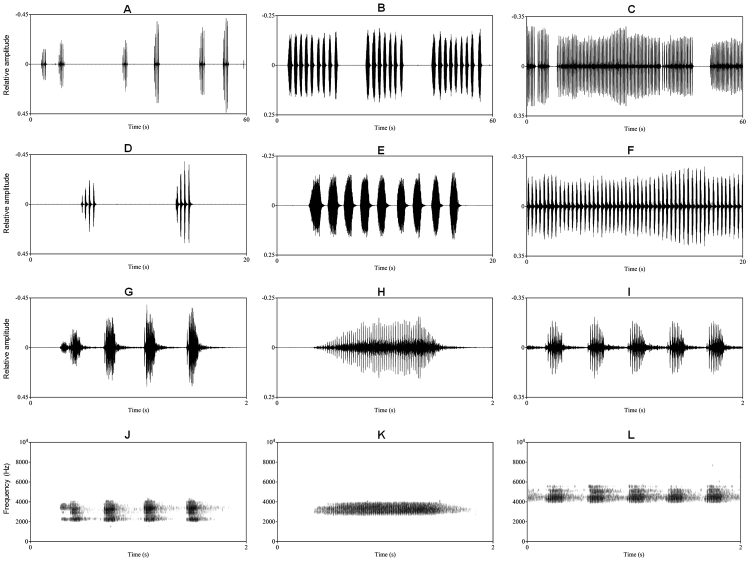
Visualization of advertisement calls of three *Megophrys* species from Medog. **A–C** visualizations of 60 seconds waveform of relative amplitude over time for *M. medogensis* (one unvouchered individual recorded in the vicinity of Medog urban area), M. cf. pachyproctus (CIB022017061807), and *Megophrys yeae* sp. nov. (paratype CIB022017061804), respectively **D–F** visualizations of 20 seconds waveform of relative amplitude over time **G–I** visualizations of two seconds waveform of relative amplitude for the species, respectively **J–L** visualizations of two seconds waveform of spectrogram for the species, respectively.

**Bioacoustics comparisons.** The advertisement calls of *Megophrys yeae* sp. nov., M. cf. pachyproctus, and *M. medogensis* were obviously different (Fig. 4; Tables 4; Suppl. material 1: Table S3). *Megophrys yeae* sp. nov. vocalizes continuous fast short calls in high-frequency, *M. medogensis* vocalizes sparse relatively deep calls in moderate speed, and as for M. cf. pachyproctus, the calls are moderate in frequency and repetition rate, but distinctly longer (call duration 491–889 ms) than the former two species (Fig. 4; Tables 4; Suppl. material 1: Table S3).

**Table 4.** Comparisons of advertisement calls between three *Megophrys* species in Medog. P-value is resulted from Mann-Whitney *U* test on each character between species. Significant level at 0.05 (* P-value < 0.05). Abbreviation for species names: MCP, M. cf. pachyproctus; MY, *Megophrys yeae* sp. nov.; and MM, *M. medogensis*.

Call character	MCP	MY	MM	P-value		
	Mean ± SD (range)	Mean ± SD (range)	Mean ± SD (range)	MH vs. MY	MH vs. MM	MY vs. MM
**Number of individuals**	6	3	3	/	/	/
**Total number of calls analyzed**	3.2 ± 2.6 (1–8)	5.0 ± 2.6 (2–7)	5.3 ± 3.5 (2–9)	/	/	/
**Call repetition rate (calls/s)**	3.0 ± 0.7 (1.9–4.1)	0.9 ± 0.2 (0.7–1.1)	1.2 ± 0.9 (0.6–2.2)	0.024*	1	0.048*
**Calls/call group**	68.9 ± 46.7 (10.3–109.3)	10.8 ± 3.3 (7.1–13.3)	4.3 ± 1.6 (2.8–6.0)	0.229	0.1	0.057
**Call duration (ms)**	139 ± 39 (99–212)	746 ± 221 (491–889)	176 ± 61 (121–241)	0.024*	0.1	0.381
**Intercall interval (ms)**	218 ± 81 (146–370)	580 ± 122 (493–720)	205 ± 514 (153–254)	0.024*	0.1	0.905
**Pulses/call**	9.2 ± 0.6 (8.5–9.9)	42.1 ± 2.0 (40.6–44.4)	17.1	0.024*	0.5	0.286
**Dominant frequency (kHz)**	4.7 ± 0.3 (4.4–5.2)	3.2 ± 0.1 (3.2–3.3)	2.5 ± 0.1 (2.4–2.6)	0.024*	0.1	0.024*
**Temperature (°C)**	17–25	17–21	19–20	/	/	/

**Figure 5.**
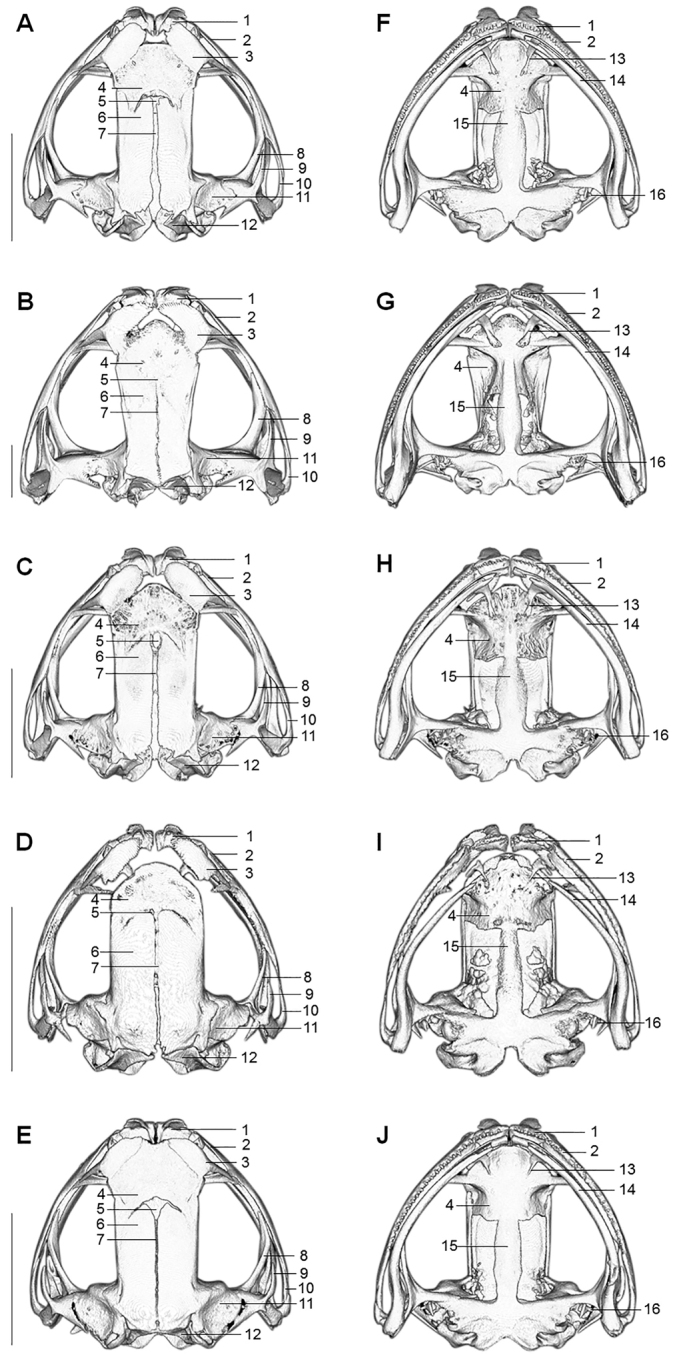
Skull of *Megophrys* species in Medog. **A–E** dorsal views of adult male holotype NWIPB770650 of *M. pachyproctus*, adult male topotype CIB022017061406 of *M. medogensis*, adult male CIB022017061805 of M. cf. pachyproctus, adult male holotype CIBMT171053 of *Megophrys zhoui* sp. nov., and adult male holotype CIB201706MT02 of *Megophrys yeae* sp. nov., respectively **F–J** ventral views of the specimens, respectively. Key to skull: 1 premaxillary; 2 maxillary; 3 nasal; 4 sphenethmoid; 5 anterior fontanelle; 6 frontoparietal; 7 sagittal suture; 8 pterygoid; 9 squamosal; 10 quadratojugal; 11 prootic; 12 exoccipital; 13 vomerine ridge; 14 mandible; 15 anterior process of parasphenoid; 16 columella auris. Scale bar equal to 5 mm.

**Skull comparisons.** Skulls of the four toad species in Medog were different on many aspects (Fig. 5; Suppl. material 1: Table S5). In general, the skulls of these five species are weakly ossified except for *M. medogensis*. Skulls of them differ from each other on the following characters: premaxillary and maxillary teeth, nasal bones contact with sphenethmoid or not, texture and shape of sphenethmoid, the shape of frontoparietal, opening of anterior fontanelle and sagittal suture, front part of anterior process parasphenoid, relatively position of exoccipitals with the line connecting conjunctions of quadratojugal and mandible, and columella auris (Fig. 5; Suppl. material 1: Table S5).

### Taxonomic accounts

#### Megophrys pachyproctus

Taxon classificationAnimaliaAnuraMegophryidae

Huang, 1981

8E13D6E4-1FCC-5815-B365-4861E119A3E3

Figs 3A, F, K, P, U , 5A, F , Suppl. material 2 : Fig. S5C; Tables 1 , Suppl. material 1: Tables S1, S2, S5

Megophrys pachyproctus Huang, 1981^*^ in Huang & Fei, 1981: 211–212. Holotype: NWIPB 770650, by original designation. Type locality: Gelin, Medog (29°11'N, 95°10'E), Xizang, China; altitude 1530 m, China.

##### Specimens examined.

Holotype: adult male NWIPB 770650.

##### Description of holotype.

(Fig. 3A, F, K, P, U; Suppl. material 1: Table S1). Measurements in mm. Adult male. Body moderate, SVL 35.7; a large swollen arc-shaped protuberance present on vent beyond cloaca and visible on both dorsal and lateral view, its length 6.8 (measured dorsally), width 4.7, and thickness 2.7.

Head wider than long (HW/HL 1.13); snout blunt in dorsal view, obtusely protruding beyond mandible in lateral view; rostral appendage absent; canthus rostralis well developed, loreal region concave; dorsal surface of snout slightly concave; nostril oval, slightly closer to snout than eye (EN/SN 1.04); eyes lager than twice tympanum (EL/TYD 2.24); eye-tympanum distance smaller than tympanum diameter (TYE/TYD 0.86); tympanum oval, obliquely orientated, upper 1/3 concealed with supratympanic ridge; interorbital space flat, wider than upper eyelids (UEW/IUE 0.89); pineal ocellus not visible; vomerine ridges well developed, acutely angled, enlarged at ends where bearing several vomerine teeth; maxillary teeth present; tongue notched posteriorly, medial lingual process absent.

Forearm moderately long and wide; fingers long and thin, without webbing and lateral fringes; subarticular tubercles absent; inner and outer metacarpal tubercles small and oval, weakly connected at lower half; finger relative lengths I < II < IV < III; base of finger I strong, larger than base of finger II; tips of fingers slightly swollen and rounded (FIIIW 0.8), without pads.

Hindlimbs relatively thin and long; thighs ca. equal length of shanks and feet; toes long and thin, relative lengths I < II < V < III < IV; tips of toes rounded; toes rudimentary webbed; lateral fringes narrow; continuous dermal ridges present under toes; outer metatarsal, and subarticular tubercles absent; inner metatarsal tubercle distinct, rounded, separate from base of toe I at a distance nearly twice its diameter; tips of toes rounded.

Dorsal surface of head and body relativity rough, densely scattered with small granules; temporal region and upper corner of mandible scattered small granules; tympanum border slightly raised; upper eyelid without pointed edge; supratympanic ridges extend from posterior upper eyelid border to region above forearm insertions, not curving above tympanum, rear part thicker than front; flanks densely covered with small granules and scattered several larger tubercles; two longitudinal ridges on dorsolateral body distinct, nearly parallel, extending from above shoulder to nearby groin; parietoscapular-sacral ridges forming a “> <” configuration, composed by rows of small tubercles, dorsal surface of forearm thighs and shanks with several rows of small tubercles transversely arranged; dorsal upper arm and other dorsal surfaces of hindlimbs covered with dense small granules; ventral surface of body and limbs smooth; pectoral glands small (diameter 0.8) and rounded, close to axilla on chest; femoral glands small (diameter 1.0) and rounded, closer to outer edge of knee than to cloaca.

##### Coloration of holotype in preservative.

(Fig. 3A, F, K, P, U). Dorsal and lateral surface of body, dorsal surface of head mostly tan; a brown triangle present between eyes, little lighter in center, anterior corners reach to near out edge of upper eyelids; indistinct “X”-shaped markings on dorsum, with small tubercles in center; darker brown stripe along with dorsolateral ridges; tubercles on flanks white, edged with dark patches; lateral surface of head tan with brown stripes radiating from orbit to upper mandible and upper eyelid; iris dark brown; a brown stripe extending from posterior corner of orbit under former half supratympanic ridges to behind tympanum, a clear thin dark stripe under edges of supratympanic ridges after tympanum, no long white stripe present on upper lip; dorsal and lateral surface of limbs mostly tan, two broad brown transverse bands on forearms, and four thin indistinct transverse bands on dorsal thighs and shanks; dorsal tarsal pale gray with three indistinct transverse bands, outer three fingers with tan blotches; gular region and chest dusty tan with a short longitudinal brown stripe in middle of throat; two light patches on edges of jaw corresponding anterior corners of eyes and front edge of brown stripe at posterior end of jaw; and one brown stripe extending from posterior end of jaws to base of forearms on both sides; abdomen dusty tan, with a dozen darker patches on middle and upper abdomen, large longitudinal dark patches present on ventral lateral abdomen hardly present; ventral surface of forelimbs and hindlimbs dusty tan mottled with light patches; pectoral and femoral glands light tan.

##### Coloration of holotype in life.

According to Huang and Fei (1981): dorsum brown or dark brown; two to four dark colored transverse bands present on forearms; and four to five dark colored transverse bands present on thighs and shanks; places around cloaca, groin, and anterior, posterior, ventral thigh orange; tips of fingers and toes light red; ventral surfaces of tarsi, metatarsus, and toes grayish brown or black-brown; lateral and ventral surface with lots of grayish black spots; a longitudinal short grayish black stripe present on middle throat; granules on dorsal surface of body and limbs light red.

##### Skull.

(Fig. 5A, F). Description based on scan of the holotype. Skull weakly ossified, width 1.12× of length; maxillary overlapping with the quadratojugal; premaxillary and maxillary teeth strong, most tooth closely positioned with others, 9/9 teeth present on left/right premaxillary, no teeth present on mandible; vomerine ridge well developed, two vomerine teeth present on enlarged posterior end of each vomerine ridge; nasal process of premaxilla protruding beyond skull; nasal bones separated from each other, inner edge mostly contact with sphenethmoid; sphenethmoid relatively smooth with few small pits on both dorsal and ventral surface, the middle one third of front edge not contacting nasal bones and truncate, separated from premaxilla; frontoparietal divided by a distinctly opening sagittal suture, sagittal suture slightly wider posteriorly; anterior fontanelle small, only slightly wider than sagittal suture; front and rear part of frontoparietal almost equally wide; posterior edge of exoccipitals posterior to the line connecting conjunctions of quadratojugal and mandible; pterygoid moderate; anterior process of squamosal slender and sharp, tip closer to the junction of pterygoid and quadratojugal than its base, posterior process present; prootic relatively smooth, separated from exoccipitals; anterior process of parasphenoid in shape of fusiform, anterior part not raised above sphenethmoid, conjunction of parasphenoid anterior process meet with sphenethmoid moderate, width ca. three quarters of the constriction near its base; columella auris short.

##### Variations.

See for morphometric variation within the three types (two adult males and one adult female) in Suppl. material 1: Table S1. According to the photo of dorsal view of the only adult male paratype NPIB 770651 presented by Fei and Yei (2016), the adult male paratype resemble the holotype in general, also has a distinct swollen arc-shaped protuberance present on end of body beyond cloaca, but different in color on dorsal body darker, and not having distinguishable “X”-shaped markings on dorsum. The adult female allotype NPIB 770652 do not have a distinct projection on vent, and the coloration on dorsal and ventral surface of body lighter than males.

##### Secondary sexual characters.

Male with gray nuptial pad on inner side of the first finger, spines on nuptial pad dense and small; single subgular vocal sac; vocal sac opening small, slit like; a distinct fatty swollen rounded projection present on the end of body beyond cloaca.

##### Distribution and natural history.

According to Huang and Fei (1981), this species was first collected at elevation 1530 m in the type locality, Gelin, Medog, Xizang, China; two adult males were found on shrubs emitting continuous calls sounds like “gazhi...gazhi...gazhi...” to the human ear; the female was found on the road near a brook.

##### Comparisons.

Megophrys pachyproctus differs from all other known congeners except M. koui and M. caudoprocta by having a distinct protuberance above vent, and further differs from the latter two species in protuberance above vent being swollen and arc-shaped (vs. not). For comparisons with subsequent undescribed species covered in this paper, refer to relevant morphological comparison sections for those species.

##### Remarks.

Megophrys pachyproctus was originally described by Huang and Fei (1981) with description and figures of the holotype, measurements of types, secondary sexual characters, and brief natural history. And then were translated into English and measurements of snout length, internasal space, interorbital space, eyelid width, diameter of eye, tympanum, and tibia width were supplemented by Huang et al. (1998). Fei et al. (2009) provided illustration of the holotype. Fei et al. (2010) and Fei and Ye (2016) provided colored drawings of the holotype and the paratype (NPIB) 770651, and colored photos of dorsal and lateral views of one living topotype from Medog (photographs by Ke Jiang). The topotype possess expanded fingertips with small disk and two large longitudinal dark patches on ventral lateral abdomen (while the holotype of M. pachyproctus does not have these character), and not having a distinct swollen arc-shaped protuberance present above vent (while the holotype of M. pachyproctus possess). Li et al. (2010) provided similar photos of a living specimen and measurements of seven specimens from Maniwong and Yarang, Medog, under the name M. pachyproctus. The body length of these specimens ranges from 26.1 mm to 27.9 mm, and Li et al. (2010) cited the body length of the male types of M. pachyproctus as 25.3 mm to 36.2 mm, which should be 35.3 mm to 36.2 mm (Huang and Fei 1981). These specimens with small body size, expanded fingertips with small disk, and two large longitudinal dark patches on ventral lateral abdomen turn out to be mostly similar with Megophrys yeae sp. nov. (see description of Megophrys yeae sp. nov.). We suggest reexamination of these specimens should be taken. Saikia and Sinha (2018) reported M. pachyproctus from Southern Xizang (27.547681°N, 93.897555°E, 1855 m), provided description, measurements (body length 37.8), and a photo of dorsal view of the single male voucher specimen V/A/NERC/1352. But the photo does not present an arc-shaped swelling above vent (Saikia and Sinha 2018: fig. 3A) as the holotype. We suggest further examination should be made to confirm the identification of the specimen. This species was also reported new range in Lao Cai and Ha Tinh Province, Vietnam (Orlov et al. 2002; Nguyen et al. 2005). Since Megophrys inhibits astonishing cryptic species biodiversity, and species like M. pachyproctus thought to be widespread from Southwest China to Vietnam confirmed to be another species (Chen et al. 2016; Tapley et al. 2017; Liu et al. 2018), records from Vietnam where more than 1000 km from type locality should be questioned and specimens should be reexamined.

#### Megophrys medogensis

Taxon classificationAnimaliaAnuraMegophryidae

Fei et al., 1983

62F126D0-9BCB-56D5-8848-A748763CDC37

**Figure 6.**
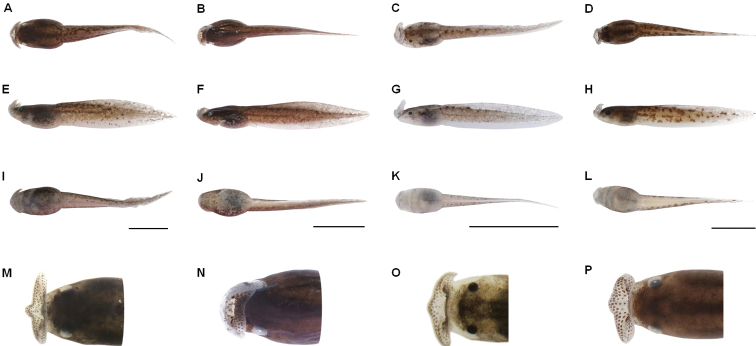
Tadpole specimens of four groups of three Megophrys species from Medog. **A–D** dorsal views of the low-middle-elevation tadpole CIBMT20170621 of M. medogensis (Goser stage 35), the high-elevation tadpole CIBMT171001 of M. medogensis (stage 27), tadpole CIBMT20170611 of M. cf. pachyproctus (stage 25), tadpole CIBMT170604 of Megophrys yeae sp. nov. (stage 35), respectively **E–H** lateral views of the tadpoles, respectively **I**–**L** ventral views of the tadpoles, respectively. M–P dorsal views of head of the tadpoles, respectively. Scale bar for body view equal to 10 mm, and for head view 2 mm.

Figs 3B, G, L, Q, V , 4A, D, G, J , 5B, G , 6A, B, E, F, I, J, M, N , 10A–C, E, F , Suppl. material 2 : Figs S1, S5A–H; Tables 1 , 2 , 3 , 4 , Suppl. material 1: Table S1-S5

Megophrys omeimontis medogensis Fei, Ye and Huang (1983)^**^: 49–52.

##### Specimens examined.

Five adult females and six adult males from Medog (Suppl. material 1: Table S1).

##### Holotype description.

Refer to Fei et al. (1983) for holotype description, Mahony et al. (2018) for picture of holotype CIB 73II0015, Fei et al. (2009) and Fei and Ye (2016) for description of coloration and picture of topotypes.

**Skull.** (Fig. 5B, G). Description based on sequenced adult male topotype CIB022017061406. Skull well ossified, width 1.21× length; maxillary overlapping with the quadratojugal; premaxillary and maxillary teeth well developed, and closely positioned with others, 11/13 teeth present on left/right of premaxillary; vomerine ridge robust; few vomerine teeth strong, present on posterior end of vomerine ridge; nasal process of premaxilla protruding beyond skull; nasal bones separated, posterior one third of inner edge contact with sphenethmoid; frontoparietal distinctly wider in front than rear; sphenethmoid relatively smooth with few small pits on dorsal and ventral surface, the middle half of front edge not contacting nasal bones and protruding forward, separated from premaxilla; frontoparietal not divided, sagittal suture occlusive; anterior fontanelle occlusive; front part of frontoparietal distinctly wider than rear; posterior edge of exoccipitals anterior to the line connecting conjunctions of quadratojugal and mandible; pterygoid robust; anterior process of squamosal slender, tip much closer to the junction of pterygoid and quadratojugal than its base, posterior process present; prootic relatively smooth, separated from exoccipitals; anterior process of parasphenoid in shape of fusiform, the front part raise above sphenethmoid from ventral view, the conjunction with sphenethmoid with width equals the constriction near base of anterior process of parasphenoid; columella slender and long.

##### Secondary sexual characters.

Adult female generally with larger body size. Average body length females 79.7 mm (n = 5, 75.7–85.5 mm), male 65.3 mm (n = 6, 63.1–68.7 mm). Males with brown nuptial pads on fingers I and II, spines on nuptial pad dense; single subgular vocal sac.

##### Tadpole.

(Fig. 6A, B, E, F, I, J, M, N; Suppl. material 1: Table S2). For low-middle-elevation tadpoles of M. medogensis, description was based on tadpole CIBMT20170621 (stage 35) which shared the same pond of sequenced tadpole CIBMT022017061808 in Bari village. They are similar on morphology. For coloration at stage 26, description based on sequenced specimen CIBMT1710101 from Yadong village. Measurements in mm. For stage 35, body 13.3, elongated; head slightly narrower than trunk, oral disk large, funnel like, 1.2× body width; three rows of short oval submarginal papillae on lower lip; middle of lower lip protruding forward, with five rounded papillae longitudinal arranged from the tip middle lower lip to oral cavity; corner of mouth with six papillae arranged in a transverse row on both sides; three transverse rows of short oval papillae on upper lip; keratodonts absent; nares closer to eyes than tip of snout (RN/NE 1.6); eyes round, positioned dorsolaterally; internarial distance (IND 3.0) 61% of the interpupilar distance (PP 4.9); spiracle mostly in left side of body, in right-handed helix from ventral view; spiracular tube not protruding beyond body wall, positioned 60% of the distance between tip of the snout and trunk-tail junction, and below the horizontal mid trunk line; tail accounts 69% of total length; dorsal fin arise above trunk-tail junction, 35% of maximum body height; ventral fin connected to the trunk, with lesser height, 27% of maximum body height; anal siphon opens medially; maximum tail muscle height 72% of maximum body height, maximum tail muscle strong, width 53% of maximum body width; 12 small curves present on both lateral side of tail muscle. For stage 26, dorsal fin arises behind trunk-tail junction. For stage 43, clear “X” and “l_l” skin ridges have present on dorsum, limbs are well developed. For high-elevation tadpoles of M. medogensis, description mostly based on sequenced tadpole CIBMT171001 (at stage 27), coloration based on sequenced tadpoles CIBMT1710106 and CIBMT1710112, collected from Gedang, Medog, Tibet Autonomous Region, China (29.463916°N, 95.769507°E, 2142 m). Body 9.5, elongated; head slightly narrower than trunk; oral disk moderate, funnel like, positioned anterior-dorsal, width equal with body width; 5 transverse rows of short oval papillae on upper lip; keratodonts absent; nares much closer to eyes than tip of snout (RN/NE 2.2); eyes round, positioned dorsolaterally; internarial distance (IND1.9) 61% of the interpupil distance (PP 3.2); spiracle barely visible from ventral view; the spiracular tube not protruding beyond body wall, positioned 63% of the distance between tip of the snout and trunk-tail junction, and below the horizontal mid trunk line; tail accounts 72% of total length; dorsal fin arise above anal siphon opens, 40% of maximum body height; ventral fin connected to the trunk, with lesser height than dorsal fin, 37% of maximum body height; anal siphon opens medially; tail muscle relatively weak, maximum height 72% of maximum body height, width only 44% of maximum body width; eleven small curves present on both lateral side of tail muscle.

Coloration of tadpoles. Low-middle-elevation tadpoles. In preservation (based on CIBMT20170621; Fig. 6A, B, E, F): dorsal body brown; lips semitransparent, papillae on lips brown; dorsal tail light brown, marbled with deep brown markings; lateral side of tail densely covered with tiny brown pigment spots, also mottled with small light colored patches, and scattered with deep brown pigments piles; fins semitransparent; ventral surface of body semitransparent, sparsely covered with brown pigments. In live: dorsal and lateral body generally in light yellow-brown; lateral side of tail mottled with light colored patches; ventral body without white patches; iris brown.

High elevation tadpoles (Fig. 6I, J, M, N). In preservation: dorsal body and tail with brown pigmentation; lateral body brown, skin coloration lighter below spiracular tube, with clear white patches; lateral tail muscle brown mottled with dense tiny white dots; upper and lower fin semitransparent brown, lower fin colored lighter; no dark patches on lateral and dorsal tail; ventral body semitransparent white, stained with light brown pigments; lips semitransparent white, papillae brown. When alive, dorsal body and tail basically deep brown, mottled with copper pigmentation, especially dense on body; lateral body brown, with cream-white patches near abdomen; lateral tail brown, scattered with tiny white pigment spots, no dark brown patches on tail; ventral surface of body semitransparent brown, covered with small white pigments; iris brown.

##### Bioacoustics.

(Fig. 4A, D, G, J; Suppl. material 1: Tables 4; Suppl. material 2: Fig. S3). A total of 16 call groups and 62 calls were analyzed. Average dominant frequency of calls low, 2.5 kHz (2.3–3.0 kHz); call repetition rate moderate, average 1.2 calls per seconds; call interval short, average 153–254 ms; call groups with average 10.8 calls; call duration long (average 746 ms), and with lots of notes (average 42.1). To the human ear, the call sound like “ga ga ga...”.

##### Distribution and natural history.

The species is currently known with certainty from the type locality in Medog County, and its distribution elevation was recorded between 680–2200 m (Fei et al. 1983, 2012, Fei and Ye, 2016; this study). This species was recorded in or near small mountain streams of tropical rain forests, sit on rocks, leaf litter, and sometimes bare soil. Calls heard between 11 June to 5 August (this study; Fei et al. 2019). Four in five females recorded during 11 June to 18 June were gravid. Males start calling before dusk under dense vegetation. Normally, two or more males call in small groups along stream banks, spacing themselves ca. 3–5 meters from each other. Sequenced tadpoles in metamorphosis were recorded on 18 June, in small mountain stream pond at 1560 m. Tadpoles of two other species of Megophrys share the same ponds. See description in following. Breeding season is supposed to including early June and may last to early August.

##### Comparison.

Refer to Mahony et al. (2018) for comparison with other species of M. major group. M. medogensis differs from M. pachyproctus by much larger body size (SVL 57.2–68.7 in 21 males vs. 35.3–35.7 in two males in the latter), absence of large protuberance above vent (vs. present in the latter), skin relatively smooth (vs. rough in the latter), frontoparietal distinctly wider in front than rear (vs. almost equally wide in the latter), sagittal suture occlusive (vs. distinctly open in the latter), and columella auris long (vs. short in the latter). For comparisons with species studied in this paper, refer to relevant morphological comparison sections for those species.

#### Megophrys cf. pachyproctus

Taxon classificationAnimaliaAnuraMegophryidae

636D8655-349B-50B5-B431-68A80A619A2E

Figs 3C, H, M, R, W , 4B, E, H, K , 5C, H , 6C, G, K, O , 7 , 10C, D, H, I , Suppl. material 2 : Figs S2, S5B, S5F; Tables 1 , 3 , 4 , Suppl. material 1: Tables S1, S2, S3, S5

Megophrys cf. pachyproctus Huang, 1981^***^

##### Specimens examined.

**Figure 7.**
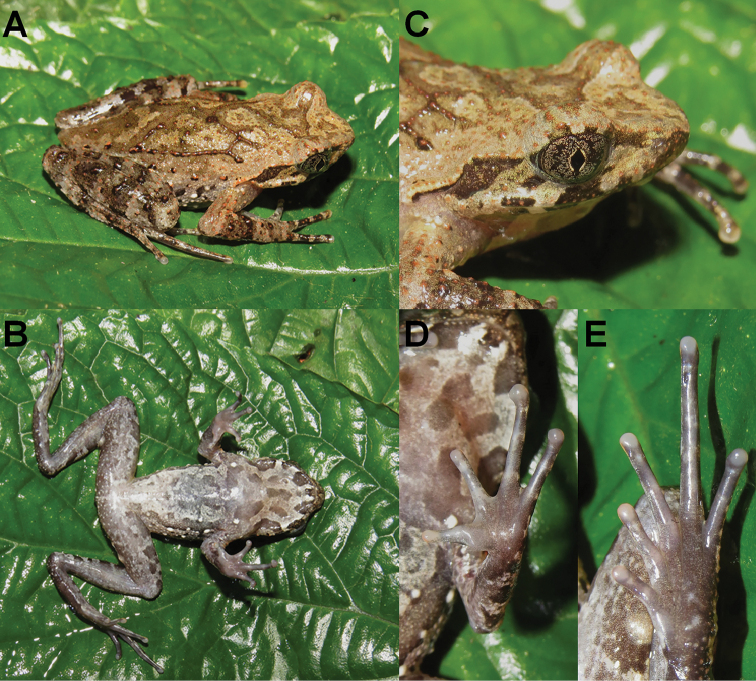
The adult male CIB022017061805 of M. cf. pachyproctus in life. **A** dorsolateral view of body **B** ventral view of body **C** dorsolateral view of head **D** ventral view of hand **E** ventral view of foot.

Four adult males, CIB022017061805 (Figs 3C, H, M, R, W, 5C, H, 7), CIB201706MT04, CIB022016061806, CIB022017061807, collected from Bari village, Medog County, Tibet Autonomous Region, China (29.32947°N, 95.36016°E, 1780 m) by SC Shi and L Ding, on 18 June 2017. One adult male (CIBMT171056), four adult females in gravidity (CIBMT171052, CIBMT171057, CIBMT171058, CIBMT171054), and one male toadlet (CIBMT171059) were collected from vicinity of Renqingbeng Temple (29.304832°N, 95.361682°E, 2003 m) by SC Shi on 26 October 2017.

##### Description of the representative (referred) specimen.

Adult male, CIB022017061805 (Figs 3C, H, M, R, W, 5C, H, 7). Measurements in mm. Body stout, relatively small size (SVL 34.8); protuberance beyond cloaca small, barely visible from ventral view, not swollen.

Head moderately large, wider than long (HW 12.3, HL 11.0, IFE 6.5, IBE 10.4); snout rounded in dorsal view, slightly projecting in profile, protruding beyond lower jaw; rostral appendage absent (SL 4.6); canthus rostralis blunt; loreal region concave, dorsal surface of snout slightly concave; nostril oval, nearly in the middle of distance from snout to eye (SN 2.2, EN 2.3), distance between nostrils almost equal to distance between upper eyelids (IN 3.9, IUE 3.8); tympanum smaller than half of eyes (EL 4.5, TYD 1.8); eye-tympanum distance subequal to tympanum diameter (TYE 1.7); tympanum irregular rounded, upper 1/3 conceal with supratympanic ridge; interorbital space flat, larger than upper eyelid (UEW 3.2); pineal ocellus not visible; vomerine ridges distinct, orientation of two ridges acutely angled, enlarged at ends where bearing several vomerine teeth; maxillary teeth present; tongue notched posteriorly, medial lingual process absent.

Forearm moderately long and wide, similar size of upper arms, shorter than hand (FAL 7.5, HAL 9.6); fingers long and thin, with rudimentary webbing; narrow lateral fringes present on finger III, indistinct on other fingers; subarticular tubercles absent; inner and outer metacarpal tubercles mostly fused, large, with the size of base of finger I; finger length formula I < II < IV < III; base of finger I strong, larger than base of finger II; tips of fingers slightly swollen, without pads (FIIIW 1.1).

Hindlimbs thin and long; tibio-tarsal articulation reaches middle eye; thighs shorter than shanks but longer than feet (TL 16.5, SHL 17.2, FOL 15.2, TFOL 24.0); toes long and thin, relative lengths I < II < V < III < IV, rudimentary webbed, with narrow lateral fringes, tips rounded, dermal ridges continuously present on ventral surface; subarticular tubercles absent; outer metatarsal tubercle tiny and rounded; inner metatarsal tubercle distinct (IMT 1.6), nearly oval, partially fused with toe I.

Skin. Dorsal surface of head and body rough, densely scattered with small granules; temporal region and upper corner of mandible with rough granules; tympanum ring slightly raised; several small granules on edges of upper eyelids; supratympanic ridges extend from posterior upper eyelids to above forearm insertions, curving above tympanum, rear part thicker than the front; skin on flanks smoother than skin on dorsum, with several large warts and lesser granules; dorsolateral ridges distinct, irregularly stretch from above shoulder to near groin; a transverse skin ridge between upper eyelids; a near “V”-shaped skin ridge between shoulders, connected with the right dorsolateral ridge by a short skin ridge, a tubercle present near the end of “V”-shaped skin ridge; two oblique skin ridges connected with dorsolateral ridges at posterior; dorsal surface of upper arm covered with small granules in three rows from shoulder to elbow; small granules on dorsum of lower arm, hand, and hindlimbs, four transverse rows of granules on thighs and shanks; ventral surface of body and limbs smooth; pectoral glands small and rounded, with the size of first fingertip, close to axilla on chest; femoral glands small, closer to outer edge of knee than to cloaca.

##### Coloration in preservative.

(Fig. 3C, H, M, R, W). Dorsal surface of head gray; dorsal surface of body pale gray; a darker gray triangle bet between eyes, little lighter in center, anterior corners reach to near out edge of upper eyelids; area around dorsal skin ridges darker, no clear “X”-shaped markings on dorsum; tubercles on flanks white and edged with dark patches on one side; lateral surface of head mottled with pale gray and grayish white; a dark stripe extend from behind upper eyelid to behind corners of the mouth, thicker in the middle, and covers tympanum entirely; no long white stripe present on upper lip; two dark strips from eyes to upper lips, two short dark bands on upper lips before eyes; iris dark covered with silver pigments radiated from pupil; dorsal and lateral surface of limbs pale gray with darker transverse bands, one or two broad dark brown transverse bands on forearms, and four indistinct transverse bands on dorsal thighs and shanks, dorsal tarsal pale gray with three indistinct transverse bands, dorsal surface of fingers and toes also covered with several darker transverse bands; gular and chest dusty white; edge of lower mandible white with five brown patches, the pair corresponding to places between nasals and eyes are largest; a short longitudinal light brown stripe present in middle of throat, two pairs of faint brown patches beside the short longitudinal light brown stripe; a brown stripes extending from posterior end of jaws to base of forearms on both sides; skin around pectoral glands faint brown, three medium size faint brown patches present on dusty white upper abdomen, lower abdomen cream-white reticulated with dusty brown pigments, no large longitudinal dark patches present on ventral lateral abdomen; ventral surface of forelimbs and hindlimbs dusty brown, mottled with several irregular brown patches; pectoral and femoral glands cream-white; nuptial pad grayish black.

##### Coloration in life.

(Fig. 7). Markings as described in preservative; flanks, lateral sides of head, dorsal surface of head, body, and limbs light brown in general with orange-red granules; tympanum and stripes under eyes brown; several cream-white dots present on flanks; two dark brown transverse bands present on forearms; four dark brown transverse bands present on thighs, shanks, and tarsi; ventral surface of head, and abdomen grayish white basically, two large longitudinal brown present on lateral sides of abdomen; chest, ventral surface of hand, thigh, and feet flesh colored; skins around cloaca, on groin, and anterior, posterior and ventral thigh without orange patches; iris dark brown reticulated with dense golden pigments, pupil edged with diamond-shaped golden ring; nuptial pad gray.

##### Skull.

(Fig. 5C, H). Skull weakly ossified, width 1.15× of length; maxillary overlapping with the quadratojugal; premaxillary and maxillary teeth weakly developed, most tooth separated from others by a distinct gap, 10/10 teeth present on left/right premaxillary, teeth absent on mandible; vomerine ridge moderate, few vomerine teeth weak, present on enlarged posterior end of vomerine ridge; nasal process of premaxilla protruding beyond skull; nasal bones separated from each other, half connected with sphenethmoid; sphenethmoid rough with curves and pits on dorsal and ventral surface, middle one third of front edge free from nasal bones, and protruding forward, separated from premaxilla; frontoparietal divided by a distinctly open sagittal suture , slightly wider posteriorly; anterior fontanelle small, slightly wider sagittal suture; front and rear part of frontoparietal almost equally wide; posterior edge of exoccipitals posterior to the line connecting conjunctions of quadratojugal and mandible; pterygoid moderate; anterior process of squamosal slender and sharp, tip closer to the junction of pterygoid and quadratojugal than its base, posterior process present; front part of prootic smooth, rear part rough, separated from exoccipitals; anterior process of parasphenoid in shape of fusiform, anterior part not raised above sphenethmoid, conjunction of parasphenoid anterior process meet with sphenethmoid narrow, width ca. half the constriction near its base; columella auris short.

##### Variation.

(Suppl. material 2: Fig. S2). The topotypes have the following differences: tympanum diameter/eye diameter ratio varies from 0.40 to 0.60 for adults, 0.36 for the juvenile CIBMT171059; skin ridges between dorsolateral ridges on dorsum vary in configuration, i.e., “> <” (CIB022016061806) and “>” (CIB022017061807); and specimen CIB022017061807 with orange-red patches on groin, and flanks stained with orange-red. The three skulls similar in morphology (e.g., premaxillary and maxillary teeth weak; the nasal bones half connected with sphenethmoid; the sphenethmoid rough and protruding forward; tip of anterior process of parasphenoid narrow), but possessing following variation: vomerine teeth only present on left vomerine ridge of CIBMT171056; sagittal suture of CIBMT171056 partially occlusive near the center.

##### Secondary sexual characters.

Adult female with larger body size, average 1.17× of males. Male with gray nuptial pad on inner first finger, spines on nuptial pad dense and small; single subgular vocal sac; vocal sac opening small, slit like; lineae musculinae absent.

##### Bioacoustics.

(Fig. 4B, E, H, K; Tables 4; Suppl. material 1: Table S3). A total of 15 call groups and 82 calls were analyzed. Average dominant frequency of calls moderate, 3.2 kHz (3.2–3.3 kHz); call repetition rate moderate, average 0.9 calls per seconds; call interval long, average 493–720 ms; call groups with average 10.8 calls; call duration long (average 746 ms), and with lots of notes (average 42.1). To human ears, sound like pebbles hitting ground continuously.

##### Tadpole.

(Fig. 6C, G, K, O; Suppl. material 1: Table S2). Description based on measurements and observation of tadpole specimen CIBMT20170611 at stage 25. Measurements in mm. Body 6.1, elongated; head slightly narrower than trunk; oral disk large, funnel like, positioned anterior-dorsal, width 1.6× of body width; 4 rows of oval submarginal papillae on middle lower lip, 3 rows of oval submarginal papillae on both sides of upper lips; all these papillae range towards oral cavity; keratodonts absent; nares much closer to eyes than tip of snout (RN/NE 3.8); eyes round, positioned dorsolaterally; internarial distance (IND1.4) 85% of the interpupil distance (PP 1.63); spiracle mostly in left side of body, in right-handed helix from ventral view, the spiracular tube not protruding beyond body wall, positioned 63% of the distance between tip of the snout and trunk-tail junction, and below the horizontal mid trunk line; tail accounts 68% of total length; dorsal fin arise above trunk-tail junction, 38% of maximum body height; ventral fin connected to the trunk, with lesser height than dorsal fin, 31% of maximum body height; anal siphon opens medially; maximum tail muscle height 72% of maximum body height, maximum tail muscle width 53% of maximum body width; eleven small curves present on both lateral side of tail muscle.

When alive, dorsal body and tail basically with yellow-brown pigmentation; two golden spots in size of eyes present on dorsolateral mid body. In preservation, dorsal body, and most part of lateral tail with brown pigmentation; ventral body and tail fin semitransparent; lateral body and tail with pigmentation, but lower fin and ventral body barely pigmented.

##### Comparison.

By having relative smaller body size (males 33.6–36.6, n = 5; females 40.6–42.8, n = 4; measurements in mm), Megophrys cf. pachyproctus differs from M. medogensis (males 57.2–68.7, n = 21), M. caudoprocta (males 70.8–81.3, n = 4); M. hoanglienensis (males 37.4–47.6, n = 11), M. jingdongensis (males 53.0–56.5, n = 3), M. liboensis (males 61.6–62.9, n = 4), M. omeimontis (males 56.0–59.5, n = 10), M. aceras (males 55.8–62.4, n = 6); M. ancrae (males 39.1–45.3, n = 8), M. damrei (male 57.1, n = 1), M. flavipunctata (males 56.9–68.4, n = 4), M. glandulosa (males 76.3–81.0, n = 10), M. himalayana (males 68.0–73.5, n = 6), M. lekaguli (males 55.6–66.6, n = 8), M. major (males 71.6–87.5, n = 12), M. mangshanensis (male 62.5, n = 1), M. maosonensis (male 77, n = 1), M. megacephala (males 45.9–53.4, n = 12), M. monticola (males 38.4–49.5, n = 17), M. periosa (males 71.3–93.8, n = 12), M. robusta (males 73.5–83.1, n = 6), M. longipes (male 47, n = 1; female 65, n = 1), M. oreocrypta (female 94.9, n = 1), M. serchhipii (male 37.1, n = 1), and M. takensis (males 47.3–53.0, n = 3).

By having relative larger body size (males 33.6–36.6, n = 5; females 40.6–42.8, n = 4; measurements in mm), Megophrys cf. pachyproctus differs from M. zunhebotoensis (male 30.0, n = 1; female 39.0, n = 1), M. rubrimera (males 26.7–30.5, n = 8), and M. angka (males 31.2–32.1, n = 2).

By tympanum present distinctly, Megophrys cf. pachyproctus differs from M. gigantica, M. nankiangensis, and M. shapingensis (vs. absent or concealed in the latter).

By vomerine ridge and teeth present, Megophrys cf. pachyproctus differs from M. wawuensis (vs. absent in the latter).

By maxillary teeth present, Megophrys cf. pachyproctus differs from M. elfina, M. gerti, M. hansi, M. koui, M. microstoma, and M. synoria (vs. absent in the latter).

By hind limbs long and head not wide and flat, Megophrys cf. pachyproctus differs from M. carinense, M. chuannanensis, M. feae, M. intermedia, and M. popei (vs. hind limbs short and head flat wide in the latter).

By lacking a single, wide and flat palpebral projection on the edge of the upper eyelid, Megophrys cf. pachyproctus differs from M. lancip, M. montana, M. parallela, M. baluensis, M. edwardinae, M. kobayashii, M. ligayae, M. nasuta, and M. kalimantanensis (vs. present in the latter).

By lacking rostral appendage, Megophrys cf. pachyproctus differs from M. stejnegeri (vs. having less rostral appendage in the latter).

By lacking a distinct horn-like tubercle at edge of upper eyelid, Megophrys cf. pachyproctus differs from M. dringi (vs. present in the latter).

By vomerine teeth present, Megophrys cf. pachyproctus differ from M. vegrandis, M. baolongensis, M. binchuanensis, M. binlingensis, M. boettgeri, M. brachykolos, M. cheni, M. kuatunensis, M. lini, M. lishuiensis, M. minor, M. obesa, M. palpebralespinosa, M. sangzhiensis, M. shuichengensis, M. spinata, M. tuberogranulata, M. wuliangshanensis, M. wushanensis, M. ombrophila, M. leishanensis, M. wugongensis, M. mufumontana, M. feii, M. auralensis, and M. huangshanensis, M. angka, M. shunhuangensis, M. jiangi, and M. xianjuensis (vs. absent in the latter).

By relatively finger lengths I < II < IV < III and nuptial pads present only on finger I, Megophrys cf. pachyproctus differs from M. nanlingensis (vs. relatively finger lengths II < I < IV < III, nuptial pads and nuptial spines invisible in males during breeding season in the latter).

By toes with rudimentary webbing, Megophrys cf. pachyproctus differs from M. serchhipii (vs. at least one fourth webbed in the latter).

By toes with narrow lateral fringes, Megophrys cf. pachyproctus differs from M. binchuanensis, M. cheni, M. jingdongensis, M. lini, M. rubrimera, M. shuichengensis, M. spinata, M. feii, M. vegrandis, and M. glandulosa (vs. wide in the latter).

By dorsal skin rough but without spines, Megophrys cf. pachyproctus differs from the following species: M. vegrandis (vs. smooth); M. medogensis (vs. smooth with small granules); M. daweimontis (vs. smooth); M. fansipanensis (vs. smooth with small granules); M. oropedion (vs. smooth with small granules); M. parva (vs. smooth); M. zhangi (vs. smooth); and M. jiulianensis (vs. dorsal skin rough with spines).

By snout rounded in dorsal view and nuptial pad only present only on finger I, Megophrys cf. pachyproctus differs from M. dongguanensis (vs. snout pointed, nuptial pads present on the first two fingers in the latter).

Megophrys cf. pachyproctus further differs from M. medogensis by the following characters: nuptial pads only present on finger I in males (vs. on the first two fingers in the latter); dorsal skin rough (vs. relatively smooth in the latter); vomerine ridge moderate, vomerine teeth weak (vs. both strong in the latter).

By having following characters of skull, Megophrys cf. pachyproctus differs from M. medogensis: skull weakly ossified, opening of anterior fontanelle present, sagittal suture distinctly open (vs. skull well ossified, opening of anterior fontanelle and sagittal suture occlusive in the latter); frontoparietal front equals rear (vs. distinctly wider in the latter); sphenethmoid rough with curves and pits, middle front edge protruding (vs. relatively smooth with few pits, truncate in the latter); exoccipitals posterior to the line connecting conjunctions of quadratojugal and mandible (vs. anterior in the latter); and columella auris short (vs. long in the latter).

By having following characters of bioacoustics, Megophrys cf. pachyproctus differs from M. medogensis (Tables 3, Suppl. material 1: Table S5): call duration significantly much longer (491–889 ms vs. 121–241 ms; P < 0.001); dominant frequency significantly higher (3.2–3.3kHz vs. 2.3–3.0 kHz; P < 0.01); and call intervals significantly longer (493–720 ms vs. 153–254 ms; P < 0.001).

Megophrys cf. pachyproctus very resemble M. pachyproctus on morphology, but differs from the latter in the following characters: protuberance beyond cloaca small, barely visible from ventral view, not swollen (vs. protuberance present on vent beyond cloaca large, swollen, arc-shaped, can be seen on both dorsal and lateral view in the latter); inner metatarsal tubercle distinct partially fused with toe I (vs. inner metatarsal tubercle separate from base of toe I at a distance nearly twice its diameter in the latter). Megophrys cf. pachyproctus further differs from M. pachyproctus by having the following characters on skull morphology: premaxillary and maxillary teeth weak, separated from others by gaps (vs. strong, closely positioned with others in the latter); inner edge of nasal bones half contact with sphenethmoid (vs. mostly in the latter); sphenethmoid rough with curves and pits, middle front edge protruding (vs. relatively smooth with few pits, truncate in the latter); and conjunction of parasphenoid anterior process meet with sphenethmoid narrow, width ca. half the constriction near its base (vs. moderate, ca. three quarters in the latter).

##### Distribution and natural history.

This group is currently known at elevation from 1560 m to 2003 m in Medog County, Tibet Autonomous Region, China. It inhabits mountain streams of subtropical forests. During June, males call on branches and leaves of bushes near mountain stream with a distance at least three meters from others, where covered with dense broad leaf forests (Figs 10C, D, H, I, Suppl. material 2: Fig. S5B, S5F). Females collected during October were gravid with well-developed eggs, and also found on leaves of floor vegetation like Elatostema species and ferns near small mountain streams. Distribution elevation overlap with M. medogensis at 1560 m, where a small stream pond was found to have tadpoles of three Megophrys species on 18 June, including M. medogensis (at stage 42), Megophrys cf. pachyproctus (at stages 26–27), and Megophrys yeae sp. nov. (at stages 28–35). Theloderma sp. and Amolops medogensis Li and Rao, 2005 were recorded at the same habitat.

#### Megophrys zhoui sp. nov.

Taxon classificationAnimaliaAnuraMegophryidae

09949423-3041-56E0-B872-AA084EB4CE15

http://zoobank.org/8E90115E-03A7-440A-9A57-60F8D8489492

**Figure 8.**
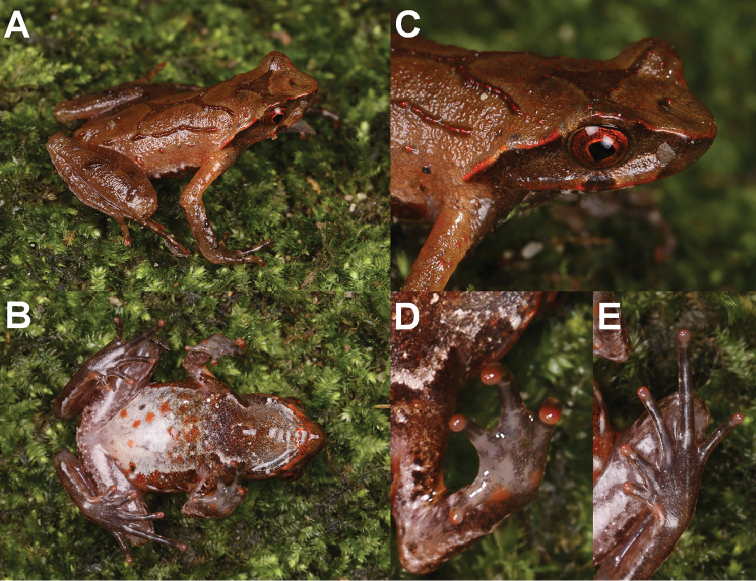
The holotype adult male CIBMT171053 of *Megophrys zhoui* sp. nov. in life. **A** dorsolateral view of body **B** ventral view of body **C** dorsolateral view of head **D** ventral view of hand **E** ventral view of foot.

Figs 3D, I, N, S, X , 5D, I , 8 , 10D, J , Suppl. material 2 : Figs S3, S5B; Tables 1 , 3 , Suppl. material 1: Tables S1, S5

##### Holotype.

(Figs 3D, I, N, S, X, 8). Adult male CIBMT171053, collected from vicinity of Renqingbeng Temple, Medog County, Tibet Autonomous Region, China (29.304832°N, 95.361682°E, 2003 m) by SC Shi on 26 October 2017.

##### Paratypes.

(Suppl. material 2: Fig. S3). Two adult gravid females CIBMT171060 and CIBMT171062, collected along with the holotype.

##### Etymology.

The specific name is in honor of Professor Zhou Kai-Ya, for his contribution to Chinese amphibian research.

##### Suggested vernacular name.

Zhou’s horned toad (English), Zhou Shi Jiao Chan (周氏角蟾, Chinese).

##### Diagnoses.

*Megophrys zhoui* sp. nov. is assigned to the genus *Megophryssensu lato* based upon molecular phylogenetic analyses and the following morphological characters: canthus rostralis well-developed; supratympanic fold distinct; axillary glands small and tit-like, on sides of the breast; head length more than 25% of body size; upper jaw protruding beyond the margin of the lower jaw; no skin fold on back of head; maxillary teeth present; tympanum distinct; hind legs long and thin.

*Megophrys zhoui* sp. nov. is distinguished from its congeners by a combination of following characters: body small (male 23.0, n = 1; females 23.5–23.9, n = 2); vomerine ridge weak, vomerine teeth absent; tympanum present, moderate; base of finger I in similar size with finger II, relative finger lengths I < II < IV < III, fingertips not expanded into small pads; toes with narrow lateral fringes or absent; inner metatarsal tubercle long oval, positioned on base of toe I; dorsal skin relatively smooth; protuberance beyond cloaca indistinct, barely visible from ventral view, not swollen; skull weakly ossified, premaxillary and maxillary teeth weak; skull wider slightly than long; nasal bones not contact with sphenethmoid.

##### Holotype description.

(Figs 3D, I, N, S, X, 8). Measurements in mm. Adult male, with well-developed testes; body slender, extremely small (SVL 23.0); protuberance beyond cloaca small, not visible from ventral view, not swollen.

Head moderate, longer than wide (HW 7.8, HL 8.3, IFE 4.5, IBE 7.2); snout near rounded in dorsal view, slightly protruding beyond lower jaw; rostral appendage absent (SL 3.6); canthus rostralis blunt; loreal region slightly concave, dorsal surface of snout slightly concave; nostril oval, closer to eye than tip of snout (SN 1.8, EN 1.4); distance between nostrils approximate distance between upper eyelids (IN 3.0, IUE 2.7); eyes twice size of tympanum (EL 2.7, TYD 1.3); pupils diamond, inferior angle slightly concave; eye-tympanum distance subequal with tympanum diameter (TYE 1.1); tympanum rounded, upper 1/3 conceal with supratympanic ridge; interorbital space flat, wider than upper eyelids (UEW 2.3); pineal ocellus not visible; two arcuate vomerine ridges present, orientation of two ridges acutely angled, not enlarged at posterior ends, shortest distance between two ridges equal to length of vomerine ridges; vomerine teeth absent; maxillary teeth present; tongue weakly notched behind, medial lingual process absent.

Forearm slender, not wider than upper arms, shorter than hand (FAL 5.2, HAL 7.1); fingers thin, without rudimentary webbing; subarticular tubercles absent; inner and outer metacarpal tubercles indistinct; base of finger I equal wide with base of finger II; finger relative length I < II < IV < III; tips of fingers slightly swollen, without pads (FIIIW 0.5).

Hindlimbs thin and long, tibio-tarsal articulation reaches middle eye; thighs shorter than shanks but longer than feet (TL 11.5, SHL 12.5, FOL10.9, TFOL 16.7); toes slender, relative length I < II < V < III < IV, rudimentary webbed, without lateral fringes, tips slightly swollen, no dermal ridges on ventral surface; subarticular tubercles absent; outer metatarsal tubercle absent; inner metatarsal tubercle long oval (IMT 1.1), positioned on base of toe I.

Dorsal surface of head and body basically smooth, with skin ridges formed by small disconnected granules; lateral surface of head smooth, tympanum ring not raised; two small granules on out edges of upper eyelid; supratympanic ridges nearly straight, extend from behind upper eyelids to above forearm insertions, rear part not thicker than the front; flanks smoother than dorsum, with several small tubercles one or two × size of nostril; skin on head scattered with tiny granules, some lager granules form a triangle between eyes; a “Y”-shaped skin ridges present between shoulders, but posterior part connected the middle of a “W”-shaped skin ridge on dorsum; several larger granules on rear dorsum behind the “W”; dorsal surface of arm smooth, scattered with tiny granules; dorsal hand and feet smooth; dorsal thighs and shanks smooth, with several larger granules; ventral surface of body and limbs smooth; pectoral glands tiny, barely visible, close to axilla on chest; pectoral glands small and rounded, slightly larger than fingertips; closer to outer edge of knee than to cloaca.

##### Coloration of holotype in preservative.

(Fig. 3D, I, N, S, X). Dorsal surface of body and limbs covered with dense gray pigments; larger granules on body and limbs light colored; a brown triangle present between eyes on head; markings on dorsum, and larger granules on dorsal thighs and shanks with brown fringes around; one broad brown transverse bands present on finger II, III and IV; two narrow transverse short bands present on lower arms; one or two faint brown transverse bands on dorsal toes. Lateral side of head pale gray mostly; skin on upper jaw between nostril and below eyes colored lighter; eyes dark with silver dense fiber around pupils and radiate on iris; supratympanic ridge light colored; chest, ventral surface of head, arms and shanks and feet covered with dense smoky gray pigments; abdomen ivory stained with smoky pigments, and scattered with several dark dots; several small ivory patches present on ventral margin of mandible; a darker brown patches with light colored inner edges extend from posterior end of jaws to ventral surface of upper arms on both sides; ventral surface hand mostly with smoky gray pigments, but base of finger I and II ivory; a brown stripe present on ventrolateral body; ventral surface of thighs smoky gray; tips of digits light colored; pectoral and femoral glands ivory.

##### Coloration of holotype in life.

(Fig. 8). Dorsal body and limbs orange-brown, granules on body orange-red; markings on dorsal body as described above; lateral head basically brown; supratympanic ridge orange; temporal region under supratympanic ridge dark brown; upper lips and canthus rostralis stained with orange; dark patches present on upper lips under eyes; iris orange-red, brighter around pupils. Flanks with several larger orange dots, ventrolateral trunk with white pigments and larger white dots. Throat, chest, arms orange-brown, mottled with dense white pigments; chin stained with orange, several small white patches present on lower lips; brown patches from posterior end of jaws to ventral surface of upper arms edged with white at inner side; upper abdomen orange-brown, stained with several faint orange dots; lower abdomen white, scattered with several clean orange dots; both lateral sides of abdomen with broad brown strips; ventral surface of thighs and shanks flesh brown, with several white tiny granules around cloaca; ventral hand with dense gray-brown pigments, base of finger I and II fleshy; inner and outer metacarpal tubercle, and tips of fingers light orange; ventral feet brown; inner metatarsal tubercle, tips of toes light orange.

##### Skull.

(Fig. 5D, J). Skull weakly ossified, width equal to length; maxillary overlapping with the quadratojugal; premaxillary and maxillary teeth weak, barely visible; teeth absent on mandible; vomerine ridge weak, vomerine teeth absent; nasal process of premaxilla protruding beyond skull; nasal bones separated from each other, completely disconnected with sphenethmoid; sphenethmoid relatively smooth with several small pits on dorsal surface and ventral surface, the front edge of sphenethmoid rounded and protruding forward, separated from premaxilla; frontoparietal partially divided by a narrowly opening sagittal suture; anterior fontanelle almost occlusive; front and rear part of frontoparietal almost equally wide; posterior edge of exoccipitals posterior to the line connecting conjunctions of quadratojugal and mandible; pterygoid moderate; anterior process of squamosal slender and sharp, tip in the middle of the distance from the base to the junction of pterygoid and quadratojugal, posterior process present; prootic relatively smooth, separated from exoccipitals; anterior process of parasphenoid in shape of narrow trapezoid, anterior part not raised above sphenethmoid, conjunction of anterior process of parasphenoid with width ca. two thirds of the constriction near the base; columella auris short.

##### Variation.

(Suppl. material 2: Fig. S3). Paratypes resemble the holotype in general but with following differences: for CIBMT171060, narrow fringes present on toes, tympanum slightly larger than half eyes, orange granules on dorsum form an “X”-shaped skin ridge and a pair of nearly parallel ridges on dorsolateral trunk, and ventral surface less brown and more orange; for CIBMT171060, a broad “X” pattern present on dorsum, and parallel skin ridges on dorsolateral trunk do not make contact with skin ridges between the left parallel ridge.

##### Secondary sexual characters.

Male with single subgular vocal sac; nuptial pad not observed in October; lineae musculinae absent.

##### Distribution and natural history.

The species is currently only discovered from type locality Renqingbeng Temple area at elevation 2003 m in Medog County, Tibet Autonomous Region, China, inhabits small streams in subtropical forests (Fig. 10D, J; Suppl. material 1: Table S5B). All three individuals were found on short herds or ferns beside small mountain stream at a distance shorter than 0.5 m to the ground (Fig. 10J). Females were found to be gravid during October, but eggs not well developed. Advertisement calls not head in October. Several gravid females of Megophrys cf. pachyproctus were collected from the same small stream with types of *Megophrys zhoui* sp. nov. on the same night. *Theloderma* sp. and *Amolops nyingchiensis* Jiang, Wang, Xie, Jiang, and Che, 2016 were recorded at the same habitat.

##### Comparison.

By body relatively smaller (male 23.0, n = 1; females 23.5–23.9, n = 2 measurements in mm), *Megophrys zhoui* sp. nov. differs from *M. pachyproctus* (males 35.3–35.7, n = 2; female 35.8, n = 1), Megophrys cf. pachyproctus (males 33.6–36.6, n = 5; females 40.6–42.8, n = 4), *M. medogensis* (males 57.2–68.7, n = 21), *M. acuta* (males 27.1–33.0, n = 10), *M. baolongensis* (males 41.8–45.0, n = 5), *M. binchuanensis* (males 32.0–36.0, n = 4), *M. binlingensis* (males 45.1–51.0, n = 3), *M. boettgeri* (males 34.5–37.8, n = 20), *M. brachykolos* (males 33.7–39.3, n = 5), *M. caudoprocta* (males 70.8–81.3, n = 4), *M. cheni* (males 26.2–29.5, n = 15), *M. daweimontis* (males 34–37, n = 18), *M. fansipanensis* (males 30.9–44.3, n = 13), *M. hoanglienensis* (males 37.4–47.6, n = 11), *M. insularis* (males 36.8–41.2, n = 5), *M. jingdongensis* (males 53.0–56.5, n = 3), *M. jinggangensis* (males 35.1–36.7, n = 2), *M. kuatunensis* (males 26.2–31.4, n = 18), *M. liboensis* (males 61.6–62.9, n = 4), *M. lini* (males 34.1–39.7, n = 20), *M. lishuiensis* (males 30.7–34.7, n = 13), *M. minor* (males 34.5–41.2, n = 4), *M. obesa* (male 35.6, n = 1; females 37.5–41.2, n = 6), *M. omeimontis* (males 56.0–59.5, n = 10), *M. palpebralespinosa* (male 36, n = 1; female 41, n = 1), *M. rubrimera* (males 26.7–30.5 n = 8), *M. sangzhiensis* (male 54.7, n = 1), *M. shuichengensis* (males 102.0–118.3, n = 7), *M. spinata* (males 47.2–54.4, n = 18), *M. tuberogranulata* (males 33.2–39.6, n = 9), *M. wuliangshanensis* (males 27.3–31.6, n = 10), *M. wushanensis* (males 30.4–35.5, n = 10), *M. ombrophila* (males 27.4–34.5, n = 5), *M. leishanensis* (males 32.1–42.3, n = 10), *M. dongguanensis* (males 30.2–39.3, n = 9), *M. nankunensis* (males 29.9–34.9, n = 11), *M. jiulianensis* (males 30.4–33.9, n = 9), *M. nanlingensis* (males 30.5–37.3, n = 10), *M. wugongensis* (males 31.0–34.1, n = 4), *M. mufumontana* (males 30.1–30.8, n = 2), *M. feii* (males 24.5–25.1, n = 4; female 28.2–28.9, n = 2), *M. vegrandis* (males 27.5–30.6, n = 4), *M. aceras* (males 55.8–62.4, n = 6); *M. ancrae* (males 39.1–45.3, n = 8), *M. auralensis* (males 76.7, n = 1), *M. damrei* (male 57.1, n = 1; female 69.1, n = 1), *M. flavipunctata* (males 56.9–68.4, n = 4), *M. glandulosa* (males 76.3–81.0, n = 10), *M. himalayana* (males 68.0–73.5, n = 6), *M. huangshanensis* (males 36.0–41.6, n = 4), *M. katabhako* (males 35.4–37.0, n = 3), *M. lekaguli* (males 55.6–66.6, n = 8), *M. longipes* (male 47, n = 1; female 65, n = 1), *M. major* (males 71.6–87.5, n = 12), *M. mangshanensis* (male 62.5, n = 1; female 73.0, n = 1), *M. maosonensis* (male 77, n = 1; female 94, n = 1), *M. megacephala* (males 45.9–53.4, n = 12), *M. monticola* (males 38.4–49.5, n = 17), *M. periosa* (males 71.3–93.8, n = 12), *M. robusta* (males 73.5–83.1, n = 6), *M. longipes* (male 47, n = 1; female 65, n = 1), *M. oreocrypta* (female 94.9, n = 1), *M. oropedion* (males 32.8–39.2, n = 7), *M. parva* (males 35.6–50.6, n = 5), *M. periosa* (males 71.3–93.8, n = 12), *M. robusta* (males 73.5–83.1, n = 6), *M. sanu* (males 39.0–46.7, n = 5), *M. serchhipii* (male 37.1, n = 1), *M. takensis* (males 47.3–53.0, n = 3), *M. zhangi* (males 32.5–37.2, n = 3), *M. zunhebotoensis* (male 30.0, n = 1; female 39.0, n = 1), *M. angka* (males31.2–32.1, n = 2), *M. shunhuangensis* (males 30.3–33.7, n = 10), *M. jiangi* (males 34.4–39.2, n = 9), and *M. xianjuensis* (males 31.0–36.3, n = 7).

By tympanum distinct moderate, larger than half eye diameter, *Megophrys zhoui* sp. nov. differs from *M. gigantica*, *M. nankiangensis*, *M. shapingensis*, and *M. wawuensis* (vs. tympanum absent, concealed or very small in the latter).

By maxillary teeth present, *Megophrys zhoui* sp. nov. differs from *M. elfina*, *M. gerti*, *M. hansi*, *M. koui*, *M. microstoma*, and *M. synoria* (vs. absent in the latter).

By hind limbs long and head not wide and flat, *Megophrys zhoui* sp. nov. differs from *M. carinense*, *M. chuannanensis*, *M. feae*, *M. intermedia*, and *M. popei* (vs. head wide flat and hind limbs short in the latter).

By lacking a single, wide and flat palpebral projection on the edge of the upper eyelid, *Megophrys zhoui* sp. nov. differs from *M. lancip*, *M. montana*, *M. parallela*, *M. baluensis*, *M. edwardinae*, *M. kobayashii*, *M. ligayae*, *M. nasuta*, and *M. kalimantanensis* (vs. present in the latter).

By lacking rostral appendage, *Megophrys zhoui* sp. nov. differs from *M. stejnegeri* (vs. having less rostral appendage in the latter).

By lacking a distinct horn-like tubercle at edge of upper eyelid, *Megophrys zhoui* sp. nov. differs from *M. dringi* (vs. present in the latter).

By vomerine ridge weak, *Megophrys zhoui* sp. nov. differs from *M. pachyproctus*, *M. medogensis*, and Megophrys cf. pachyproctus (vs. vomerine ridge stronger in the latter); differs from *M. vegrandis*, *M. baolongensis*, *M. binchuanensis*, *M. boettgeri*, *M. kuatunensis*, *M. lishuiensis*, *M. wuliangshanensis*, *M. wushanensis*, *M. ombrophila*, *M. leishanensis*, *M. feii*, *M. huangshanensis*, *M. shunhuangensis*, and *M. jiangi* (vs. absent in the latter).

By vomerine teeth absent, *Megophrys zhoui* sp. nov. differs from Megophrys cf. pachyproctus, *M. pachyproctus*, *M. medogensis*, *M. caudoprocta*, *M. daweimontis*, *M. fansipanensis*, *M. hoanglienensis*, *M. insularis*, *M. jingdongensis*, *M. jinggangensis*, *M. liboensis*, *M. omeimontis*, *M. rubrimera*, *M. dongguanensis*, *M. nankunensis*, *M. jiulianensis*, *M. nanlingensis*, *M. aceras*, *M. ancrae*, *M. damrei*, *M. flavipunctata, M. glandulosa*, *M. himalayana*, *M. katabhako*, *M. lekaguli*, *M. longipes*, *M. major*, *M. mangshanensis*, *M. maosonensis*, *M. megacephala*, *M. monticola*, *M. oreocrypta*, *M. oropedion*, *M. parva*, *M. periosa*, *M. serchhipii*, *M. takensis*, *M. zhangi*, and *M. zunhebotoensis* (vs. present in the latter).

By toes with narrow lateral fringes or absent, *Megophrys zhoui* sp. nov. differs from *M. binchuanensis*, *M. cheni*, *M. jingdongensis*, *M. lini*, *M. rubrimera*, *M. shuichengensis*, *M. spinata*, *M. feii*, *M. vegrandis*, and *M. glandulosa* (vs. wide in the latter).

By dorsal skin relatively smooth, *Megophrys zhoui* sp. nov. differs from *M. pachyproctus*, Megophrys cf. pachyproctus, *M. insularis*, *M. jinggangensis*, *M. tuberogranulata*, *M. wuliangshanensis*, *M. leishanensis*, *M. dongguanensis*, *M. jiulianensis*, *M. nanlingensis*, *M. wugongensis*, *M. mufumontana*, and *M. feii* (vs. rough in the latter).

By tympanum moderate (TYD/EL 0.40–0.60, n = 9), *Megophrys zhoui* sp. nov. differs from species with large tympanum: *M. brachykolos* (0.70–0.75, n = 7); *M. jinggangensis* (0.73–0.88, n = 5), and *M. takensis* (0.71–0.77, n = 4).

By fingertips not expanded into small pads, *Megophrys zhoui* sp. nov. differs from *M. vegrandis*, *M. ancrae*, and *M. feii* (vs. fingertips with small pads in the latter).

By the following characters, *Megophrys zhoui* sp. nov. differs from *M. pachyproctus*: protuberance beyond cloaca small, not visible from ventral view, not swollen (vs. protuberance present on vent beyond cloaca large, swollen, arc-shaped, visible on both dorsal and lateral view in the latter); and inner metatarsal tubercle long oval, positioned on base of toe I (vs. inner metatarsal tubercle rounded, separate from base of toe I at a distance nearly twice its diameter in the latter).

By having following differences on skull morphology, *Megophrys zhoui* sp. nov. differs from *M. pachyproctus*: premaxillary and maxillary teeth weak, barely visible or separated from others by gaps (vs. strong, closely positioned with others in the latter); nasal bones not contact with sphenethmoid (vs. mostly in the latter); and middle front edge of sphenethmoid protruding (vs. truncate in the latter).

By base of finger I in similar size with finger II, relative finger lengths I < II < IV < III, *Megophrys zhoui* sp. nov. differs from *M. medogensis* (vs. base of finger I distinctly larger than finger II, relative finger lengths II < I < IV < III in the latter).

By having following differences on skull, *Megophrys zhoui* sp. nov. differs from *M. medogensis*: skull weakly ossified, opening of anterior fontanelle present, sagittal suture narrowly or wide open (vs. skull well ossified, opening of anterior fontanelle and sagittal suture occlusive in the latter); premaxillary and maxillary teeth weak, barely visible or separated from others by gaps (vs. strong, closely positioned with others in the latter); frontoparietal front equals rear (vs. distinctly wider in the latter); exoccipitals posterior to the line connecting conjunctions of quadratojugal and mandible (vs. anterior); and columella auris short (vs. long in the latter).

By base of finger I similar in size with finger II, nasal bones not in contact with sphenethmoid, and texture of sphenethmoid relatively smooth with several small pits, *Megophrys zhoui* sp. nov. differs from Megophrys cf. pachyproctus (vs. base of finger I larger than the base of finger II, nasal bones mostly contact with sphenethmoid, and sphenethmoid rough with curves and pits in the latter).

#### Megophrys yeae sp. nov.

Taxon classificationAnimaliaAnuraMegophryidae

A1D67614-55C0-5A09-A338-9B41D1E1934F

http://zoobank.org/983FA221-7721-49AE-B8F7-568383A19D18

Figs 3E, J, O, T, Y , 4C, F, I, L , 5E, J , 6D, H, L, P , 9 , 10A, C, G , Suppl. material 2 : Figs S4, S5A, S5B, S5E, S5F; Tables 1 , 2 , 3 , 4 , Suppl. material 1: Tables S1-S3, S5

##### Holotype.

**Figure 9.**
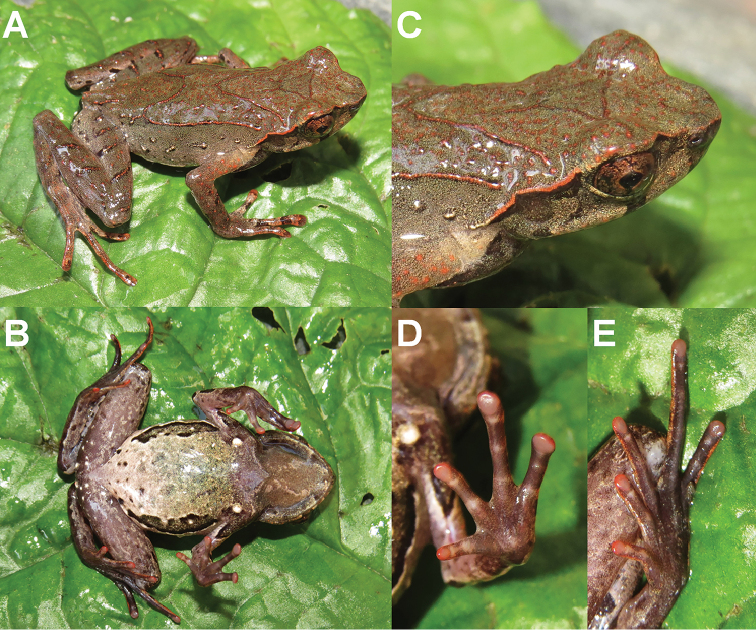
The holotype adult male CIB201706MT02 of *Megophrys yeae* sp. nov. in life. **A** dorsolateral view of body **B** ventral view of body **C** dorsolateral view of head **D** ventral view of hand **E** ventral view of foot.

(Figs 3E, J, O, T, Y, 4C, F, I, L, 9). CIB201706MT02, adult male, collected in Beibeng village, Medog County, Tibet Autonomous Region, China (29.24292°N, 95.18561°E, 870 m), at 1:40 h on 15 June 2017 by SC Shi and L Ding.

##### Paratypes.

Thirteen specimens (eleven males and two females) from Medog County, Tibet Autonomous Region, China. Four adult males (CIB201706MT01, CIB022017061102, CIB022017061103, and CIB022017061104) collected in Didong village (29.22508°N, 95.12463°E, 670 m) on 11 June 2017 by SC Shi and L Ding. One adult female (CIB201706MT03) collected on 13 June 2017 in Medog urban neighborhood (29.32213°N, 95.31324°E, 907 m) by SC Shi and L Ding. One adult female (CIBMTXC-201701-043) and one adult male (CIBMTXC-201701-044) collected on 28 May 2017 in Medog City neighborhood by F Xie and DW Yang. Two adult males (CIB022017061606 and CIB022017061407) collected in the same location of holotype by SC Shi and L Ding. One male (CIB022017061804) collected in Bari village (29.32947°N, 95.36016°E, 1780 m) at 21:01 18 June 2017 by S.C. Shi. Two adult males (CIBMT171065 and CIBMT171066) collected on 10 and 24 October 2017 in Yarang village (29.29485°N, 95.28126°E, 795 m) by F Xie and DW Yang. One adult male (CIBMT171064) collected at 23:54, 25 October 2017 in Yadong village in the vicinity of Medog city suburb (29.32654°N, 95. 34397°E, 1073 m) by SC Shi and B Wang.

##### Etymology.

The specific name *yeae* is in honor of Professor Ye Chang-Yuan, for her contribution to Chinese amphibian research and inspiration for younger generations of Chinese herpetologists.

##### Suggested vernacular name.

Ye’s horned toad (English), Ye Shi Jiao Chan (叶氏角蟾, Chinese).

##### Diagnoses.

*Megophrys yeae* sp. nov. is assigned to the genus *Megophryssensu lato* based on molecular phylogenetic analyses and the following morphological characters: canthus rostralis well-developed; a tiny horn’-like tubercle at edge of upper eyelid present; supratympanic fold distinct; axillary glands small and tit-like, on sides of the breast; oral disc of tadpoles funnel-like; mouth of tadpoles lacking transverse rows of teeth; head length more than 25% of body size; upper jaw protruding beyond the margin of the lower jaw; no skin fold on back of head; maxillary teeth present; tympanum distinct; hind legs long and thin.

*Megophrys yeae* sp. nov. is distinguished from its congeners by a combination of following characters: body relatively small (males 23.8–29.1 mm, n = 12; females 27.9–31.3 mm, n = 2); vomerine ridge weak, vomerine teeth absent; base of first finger weak, size equal to the base of second finger, tips of fingers II-IV flat, expand to small pad; foot of males shorter (FOL 10.8–12.6 mm, n = 12); dorsal skin being relatively smooth; protuberance beyond cloaca small, not visible from ventral view, not swollen; nuptial pad absent; skull weakly ossified, wider than long; premaxillary and maxillary teeth weak, separated from others by gaps; texture of sphenethmoid smooth, without curves and pits; anterior fontanelle opening large, sagittal suture occlusive; advertisement call short and fast (duration 99–212 ms, repetition rate 1.9–4.1 call/s, intervals, n = 6), and dominant frequency high (4.4–5.2 kHz, n = 6).

##### Description of holotype.

(Figs 3E, J, O, T, Y, 4C, F, I, L, 9). Measurements in mm. Adult male. Body small and slender (SVL 27.5); protuberance beyond cloaca small, not visible from ventral view, not swollen.

Head moderate, wider slightly than long (HW 9.8, HL 9.0, IFE 5.1, IBE 8.7); snout rounded in dorsal view, slightly projecting in profile, protruding beyond lower jaw, rostral appendage absent (SL 3.6); loreal region vertical and concave; canthus rostralis blunt; dorsal surface of snout slight concave; nostrils oval, nearly in the middle of distance from snout to eye(SN 1.9, EN 2.0); distance between nostrils (IN 3.2) almost equal with the shortest distance between upper eyelids (IUE 3.1); tympanum small, rounded, diameter (TYD 1.6) less than half of eye length (EL 3.8 mm), upper one third of tympanum anulus merge with supratympanic fold (Figure 5C); eye-tympanum distance (TYE 1.6) equal to tympanum diameter; pupil near oval, with a gap at lower edge; visible pineal ocellus absent; vomerine ridges weak, interval longer than its length, vomerine teeth absent; tongue feebly notched behind, hardly visible, with no medial lingual process.

Forearm long and slim, forearm length (FAL 7.0) 25% of body length, slightly shorter than hand (HAL 8.3), not enlarged relative to the upper arm; relative finger lengths I < II < IV < III; base of first finger weak, size equal to the base of second finger; tips of finger I rounded, slightly swollen, tips of fingers II-IV flat and expanded, forming small oval pads (FIIIW 1.2, FIVW 1.3), pads without grooves and distinctively larger than terminal phalanges; fingers rudimentary webbed, with ventral callous ridges and narrow lateral fringes; subarticular and supernumerary tubercles absent, palmar tubercles indistinct.

Hindlimbs long and thin, tibio-tarsal articulation reaches area between nostril and eye; heels meet when thighs are positioned at right angles to the body, shank (SHL 14.1) slightly longer than thigh (TL 12.3) and feet (FOL 12.5, TFOL 19.7); toes thin, rudimentary webbed, with ventral callous ridges and narrow fringes; relatively toes lengths I < II < V < III < IV; tips of toes flat and slightly dilated, without grooves, slightly larger than terminal phalanges; inner metatarsal tubercle weak (IMT 1.9) and elliptical, outer metatarsal tubercle, subarticular and supernumerary tubercles absent.

Dorsal body and head relatively smooth, with tiny tubercles scattered on dorsal part of body and limbs; tiny tubercles on most of dorsum form a large “W” skin ridge from behind supratympanic fold curve to ca. one third distance left of groin, a “V” between shoulders ahead of “W”, and a triangle between eyes; edges of snout, eyelids, especially supratympanic fold and flanks scattered with larger tubercles; supratympanic fold thin, extend from rear of eyelid, curves down above tympanum to shoulder; small tubercles on dorsal thigh and shank arranged in several transversal rows. Ventral surface of body smooth; a granular line present on ventrolateral side of belly, interrupted on left side; several small glandular tubercles present around cloaca; pectoral glands small, as large as tips of finger II, raised slightly, close to axilla; single femoral gland on ventral thigh small and slightly raised, closer to knee than cloaca.

##### Coloration of holotype in life.

(Fig. 9). Dorsal head and body light brown, tiny tubercles scattered on dorsum and head orange-red; skin ridges edged with faint brown, forming a barely visible “X” pattern on dorsum and triangular on head; skin ridges on thigh edged with narrow black-brown; supratympanic fold orange-red; one short black vertical bar on each side of upper lips beneath the eyes; tympanum pale gray; a black streak under supratympanic fold; tubercles on flanks edged with small dark blotches; two thin ambiguous transverse dark band on dorsal forearms; fingers II-IV with transverse dark band on dorsal surface; dorsal surface of fingers and toes colored with orange-red; flanks light brown; throat pale dusty gray; chest mottled gray stained with light purple between axillary glands; two pale gray streaks from lower place of joins of jaws extend to half of ventral upper arm; abdomen ivory, mottled gray on upper part, several small dark dots scattered rear; a large dark streaks present on both lateral sides of abdomen, from behind axilla to near groin, bordering the creamy white ventrolateral granular line on belly; groin not colored with red; ventral surfaces of thighs light purple mottled with tiny smoky white pigments; small glandular tubercles around cloaca and ivory; ventral surface of shanks and arms with large dark patches; palm and ventral surfaces of foot purplish gray; tips of digits orange edges; pectoral glands and femoral glands creamy white; iris orange-bronze.

##### Coloration of holotype in preservative.

(Fig. 3E, J, O, T, Y). Dorsal body gray, triangle on head and “X” pattern on dorsum barely visible; tubercles on dorsum, dorsal surface of head and limbs light gray; tympanum brown, a black streak under supratympanic fold; vertical bar beneath the eyes pale gray; tubercles on flanks edged with small black blotches; tubercles rows on dorsal thigh and shank with dark edges more or less; dorsal surface of forelimbs and hindlimbs dark gray with several ambiguous transverse dark bands; fingers II, III and IV with transverse dark band on dorsal surface; tips of digits light colored; throat, chest, and upper abdomen dusty gray, lower part of abdomen off-white; two dark streaks from lower place of joins of jaws extend to half of ventral upper arm; several small dark patches scattered on lower abdomen; two large dark streaks on lateral sides of abdomen; ventral surface of lower arm whitish, with a pale gray patches connected to hand; palm and ventral surface of foot dusty gray, with light colored fingertips; ventral surface of thigh dusty gray, femoral glands and glandular tubercles around cloaca white; ventral surface of shank dusty gray with several large pale gray patches; iris mottled copper.

##### Skull.

(Fig. 5E, J). Skull rather small and weakly ossified, width 1.16× of length; maxillary overlapping with the quadratojugal; premaxillary and maxillary teeth moderately developed, independent with each other, 10/7 teeth present on left/right premaxillary, teeth absent on mandible; vomerine ridge weak, vomerine teeth absent; nasal process of premaxilla protruding beyond skull; nasal bones separated, posterior edges completely contact with sphenethmoid; sphenethmoid smooth on both dorsal and ventral surface, without curves and pits, the front edge of sphenethmoid concave, in contact with premaxillary; frontoparietal not divided, sagittal suture occlusive; anterior fontanelle opening large, triangular, width approximately the same as nasal bones; the front and rear part of frontoparietal almost equally wide; posterior edge of exoccipitals posterior to the line connecting conjunctions of quadratojugal and mandible; pterygoid moderate; anterior process of squamosal slender and sharp, tip closer to the junction of pterygoid and quadratojugal than its base, posterior process present; prootic relatively smooth, separated from exoccipitals; anterior process of parasphenoid in shape of narrow trapezoid, anterior part not raised above sphenethmoid, conjunction of anterior process of parasphenoid with width approximately the same as the constriction near the base; columella auris short.

##### Variation

(Suppl. material 2: Fig. S4; Suppl. material 1: Table S1). Two female specimens with no lateral fringes on toes. A distinct brown “X” marking on dorsum and a clear triangular on head present on CIB022017061407, CIBMT171064. Abdomens of CIB201706MT01, CIB022017061103 with dark patches on both sides instead of two large streaks. Overall coloration of CIB022017061102, CIB022017061103, CIB022017061103, CIB201706MT01 lighter, without visible “X” marking on dorsum. The “W” skin ridges on dorsum of CIBMT171065 and CIBMT171066 shattered into short disconnected bars. The tympanum of CIBMT171065 not merged with supratympanic fold. The ventrolateral line varies among individuals, in some (e.g., CIB201706MT01) it is interrupted and short, in others (e.g., CIBMT171066) it is simply formed by two separated granules.

##### Secondary sexual characters.

An internal single subgular vocal sac present in male. Vocal openings present at rear of part the mouth. Calling males without nuptial pad on finger.

##### Advertisement call.

(Fig. 4C, F, I, L; Tables 4; Suppl. material 1: Table S3). A total of 19 call groups and 176 calls were analyzed. *Megophrys yeae* sp. nov. has a high dominant frequency (average 4.7 kHz, range 4.4–5.2 kHz). Calls frequent, average calls per seconds 3.0, vary from 1.9 to 4.1; average intercall interval 218 ms, vary from 649 ms to 119 ms when ambient temperatures vary from 17 °C to 25 °C. The number of calls in each call group average 68.9, range from 5 to 187. Calls short, duration average 139 ms, range from 89 ms to 246 ms. Pules per call average 9.2, vary from 7 to 12. To human ears, sound like cricket.

##### Tadpole.

(Fig. 6D, H, L, P; Suppl. material 1: Table S2). Stages 28–35. Body length range from 10.2–11.4 mm, elongated and slender; oral disk funnel like; positioned anterior-dorsal, large, width average 1.4 (1.1–1.5) × of maximum body width, with five nearly parallel rows of oval submarginal papillae on middle lower lip, three rows of oval submarginal papillae on both sides of upper lips, both submarginal papillae rows on upper and lower lips rows pointing towards oral cavity, smaller outer sides; nares oval and are closer to the eye than to the snout (RN/NE average 2.2, 1.8–2.6); internarial distance average 69% (64–78%) of the interorbital distance; eyes positioned dorsolaterally, the pupils rounded; spiracle in right-handed helix from ventral view, spiracular tube not protruding beyond body wall, positioned 53% (47–57%) of the distance between tip of the snout and trunk-tail junction, and opens laterally; the tail makes up average 69% (67–72%) of the total body length; dorsal fin arise behind trunk-tail junction, average 35% (30–41%) of maximum body height; the basal tail width average 60% (48–65%) of the maximal trunk width; keratodonts absent.

Coloration of tadpoles in life: dorsal body brown with dense copper pigments; dorsal tail brown, scattered with copper pigments; lateral tail above lower fin mottled with copper patches; ventral surface of body, and lower fin semi-transparent; iris light brown. Coloration in preservative: dorsal body brown; dorsal tail light brown scattered with brown patches; lateral sides of body brown; lateral tail semitransparent brown, muscle scattered with a lot of distinct brown patches; fins semitransparent stained with little brown, no pigments on lower fin except latter 1/3; ventral body semitransparent white, with tiny gray pigments scattered on throat and chest; ventral tail off-white; lips semitransparent white, papillae brown.

##### Distribution and natural history.

**Figure 10.**
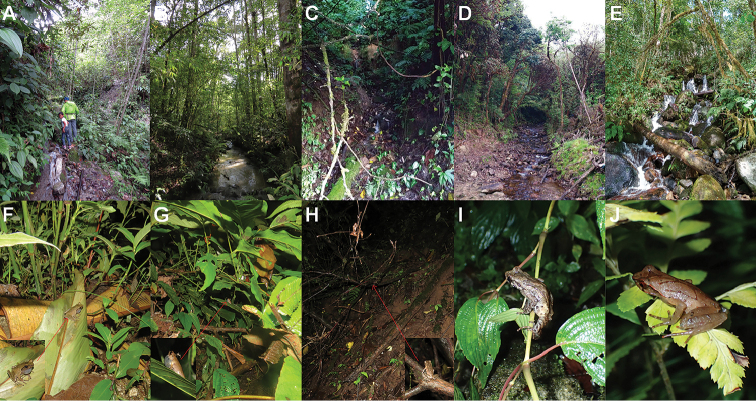
Microhabitats of *Megophrys* toads in the field in Medog. **A** stream at elevation 850 m in Didong village, harboring the low-middle-elevation *M. medogensis* and *Megophrys yeae* sp. nov. **B** a stream at 1530 m in Gelin village, hosting the low-middle-elevation *M. medogensis* and *M. pachyproctus***C** a stream at 1780 m in Bari village, harboring low-middle-elevation *M. medogensis*, M. cf. pachyproctus and *Megophrys yeae* sp. nov. **D** a stream at 2003 m in the vicinity of Renqingbeng Temple, hosting M. cf. pachyproctus and *Megophrys zhoui* sp. nov. **E** a stream at 2142 m in Gedang village, hosting *M. medogensis***F** one adult male of low-middle-elevation *M. medogensis* calling on a dead leaf on the tropical forest ground nearby a stream in Didong village **G** the adult male paratype CIB022017061102 of *Megophrys yeae* sp. nov. calling on a leaf of dense bushes under tropical forest, ca. 0.5 m above a stream in Didong village **H** the adult male CIB022017061806 of M. cf. pachyproctus calling on a branch of dead bush, ca. 0.5 m above ground under subtropical forest in Bari village **I** the gravid female CIBMT171054 of M. cf. pachyproctus precariously climbing up onto a stem of herb, ca. 0.3 m above a tiny stream under subtropical forest in Renqingbeng **J** the adult male holotype CIBMT171053 of *Megophrys zhoui* sp. nov. sitting on a split of a fern leaf in a small stream under subtropical forest in the vicinity of Renqingbeng Temple.

This species is currently known from five localities in Medog County, Tibet Autonomous Region, China (Fig. 1). All calling males recorded on June and October were found on herb leaves near or upon small stream in tropical forest (Fig. 10A, C, G; Suppl. material 2: Fig. S5A, B, E, F). Eggs in adult female (CIB201706MT03) are in two different development stage: pure yellow eggs with diameter of 1.1 mm, and semitransparent eggs with size half or less of the former. A total of 45 larger yellow eggs were counted, smaller semitransparent eggs more than 70. Thus, breeding season is suggested including June to October, and this species may lay eggs more than once during one season. The new species was recorded at elevation between 670 m to 1780 m. On 18 June 2017, four males of *Megophrys pachyproctus* (CIB201706MT04–CIB022016061806, CIB022017061807) were calling in the same stream where one male (CIB022017061804) of the new species was calling together at nearest distance ca. 3 meters in Bari Village (29.32947°N, 95.36016°E, 1780 m). From its habitat, other amphibians like *Megophrys medogensis*, Megophrys cf. pachyproctus, *Odorrana zhaoi* Li, Lu, and Rao, 2008, *Amolops medogensis*, and *Huangixalus translineatus* Wu, 1977 were also recorded.

##### Comparison.

By body relatively smaller (males 23.8–29.1, n = 12; females 27.9–31.3, n = 2; measurements in mm), *Megophrys yeae* sp. nov. differs from *M. pachyproctus* (males 35.3–35.7, n = 2; female 35.8, n = 1), Megophrys cf. pachyproctus (males 33.6–36.6, n = 5; females 40.6–42.8, n = 4), *M. medogensis* (males 57.2–68.7, n = 21), *M. baolongensis* (males 41.8–45.0, n = 5), *M. binchuanensis* (males 32.0–36.0, n = 4), *M. binlingensis* (males 45.1–51.0, n = 3), *M. boettgeri* (males 34.5–37.8, n = 20), *M. brachykolos* (males 33.7–39.3, n = 5), *M. caudoprocta* (males 70.8–81.3, n = 4), *M. daweimontis* (males 34–37, n = 18), *M. fansipanensis* (males 30.9–44.3, n = 13), *M. hoanglienensis* (males 37.4–47.6, n = 11), *M. insularis* (males 36.8–41.2, n = 5), *M. jingdongensis* (males 53.0–56.5, n = 3), *M. jinggangensis* (males 35.1–36.7, n = 2), *M. liboensis* (males 61.6–62.9, n = 4), *M. lini* (males 34.1–39.7, n = 20), *M. lishuiensis* (males 30.7–34.7, n = 13), *M. minor* (males 34.5–41.2, n = 4), *M. obesa* (male 35.6, n = 1; females 37.5–41.2, n = 6), *M. omeimontis* (males 56.0–59.5, n = 10), *M. palpebralespinosa* (male 36, n = 1; female 41, n = 1), *M. sangzhiensis* (male 54.7, n = 1), *M. shuichengensis* (males 102.0–118.3, n = 7), *M. spinata* (males 47.2–54.4, n = 18), *M. tuberogranulata* (males 33.2–39.6, n = 9), *M. wushanensis* (males 30.4–35.5, n = 10), *M. leishanensis* (males 32.1–42.3, n = 10), *M. dongguanensis* (males 30.2–39.3, n = 9), *M. nankunensis* (males 29.9–34.9, n = 11), *M. jiulianensis* (males 30.4–33.9, n = 9), *M. nanlingensis* (males 30.5–37.3, n=10), *M. wugongensis* (males 31.0–34.1, n = 4), *M. mufumontana* (males 30.1–30.8, n = 2), *M. aceras* (males 55.8–62.4, n = 6); *M. ancrae* (males 39.1–45.3, n = 8), *M. auralensis* (males 76.7, n = 1), *M. damrei* (male 57.1, n = 1; female 69.1, n = 1), *M. flavipunctata* (males 56.9–68.4, n = 4), *M. glandulosa* (males 76.3–81.0, n = 10), *M. himalayana* (males 68.0–73.5, n = 6), *M. huangshanensis* (males 36.0–41.6, n = 4), *M. katabhako* (males 35.4–37.0, n = 3), *M. lekaguli* (males 55.6–66.6, n = 8), *M. longipes* (male 47, n = 1; female 65, n = 1), *M. major* (males 71.6–87.5, n = 12), *M. mangshanensis* (male 62.5, n = 1; female 73.0, n = 1), *M. maosonensis* (male 77, n = 1; female 94, n = 1), *M. megacephala* (males 45.9–53.4, n = 12), *M. monticola* (males 38.4–49.5, n = 17), *M. periosa* (males 71.3–93.8, n = 12), *M. robusta* (males 73.5–83.1, n = 6), *M. longipes* (male 47, n = 1; female 65, n = 1), *M. oreocrypta* (female 94.9, n = 1), *M. oropedion* (males 32.8–39.2, n = 7), *M. parva* (males 35.6–50.6, n = 5), *M. periosa* (males 71.3–93.8, n = 12), *M. robusta* (males 73.5–83.1, n = 6), *M. sanu* (males 39.0–46.7, n = 5), *M. serchhipii* (male 37.1, n = 1), *M. takensis* (males 47.3–53.0, n = 3), *M. zhangi* (males 32.5–37.2, n = 3), *M. zunhebotoensis* (male 30.0, n = 1; female 39.0, n = 1), *M. angka* (males 31.2–32.1, n = 2), *M. shunhuangensis* (males 30.3–33.7, n = 10), *M. jiangi* (males 34.4–39.2, n = 9), and *M. xianjuensis* (males 31.0–36.3, n = 7).

By tympanum distinct moderate, *Megophrys yeae* sp. nov. differs from *M. gigantica*, *M. nankiangensis*, *M. shapingensis*, and *M. wawuensis* (vs. absent, concealed or very small in the latter).

By maxillary teeth present, *Megophrys yeae* sp. nov. differs from *M. elfina*, *M. gerti*, *M. hansi*, *M. koui*, *M. microstoma*, and *M. synoria* (vs. absent in the latter).

By hind limbs long and head not wide and flat, *Megophrys yeae* sp. nov. differs from *M. carinense*, *M. chuannanensis*, *M. feae*, *M. intermedia*, and *M. popei* (vs. head wide flat and hind limbs short in the latter).

By lacking a single, wide and flat palpebral projection on the edge of the upper eyelid, *Megophrys yeae* sp. nov. differs from *M. lancip*, *M. montana*, *M. parallela*, *M. baluensis*, *M. edwardinae*, *M. kobayashii*, *M. ligayae*, *M. nasuta*, and *M. kalimantanensis* (vs. present in the latter).

By lacking rostral appendage, *Megophrys*yeae sp. nov. differs from *M. stejnegeri* (vs. having less rostral appendage in the latter).

By lacking a distinct horn-like tubercle at edge of upper eyelid, *Megophrys yeae* sp. nov. differs from *M. dringi* (vs. present in the latter).

By vomerine ridge weak, *Megophrys yeae* sp. nov. differs from *M. pachyproctus*, *M. medogensis*, and Megophrys cf. pachyproctus (vs. stronger in the latter); differs from *M. vegrandis*, *M. baolongensis*, *M. binchuanensis*, *M. boettgeri*, *M. kuatunensis*, *M. lishuiensis*, *M. wuliangshanensis*, *M. wushanensis*, *M. ombrophila*, *M. leishanensis*, *M. feii*, *M. huangshanensis*, *M. shunhuangensis*, *M. jiangi*, and *M. xianjuensis* (vs. absent in the latter).

By vomerine teeth absent, *Megophrys yeae* sp. nov. differs from Megophrys cf. pachyproctus, *M. pachyproctus*, *M. medogensis*, *M. caudoprocta*, *M. daweimontis*, *M. fansipanensis*, *M. hoanglienensis*, *M. insularis*, *M. jingdongensis*, *M. jinggangensis*, *M. liboensis*, *M. omeimontis*, *M. rubrimera*, *M. dongguanensis*, *M. nankunensis*, *M. jiulianensis*, *M. nanlingensis*, *M. aceras*, *M. ancrae*, *M. damrei*, *M. flavipunctata*, *M. glandulosa*, *M. himalayana*, *M. katabhako*, *M. lekaguli*, *M. longipes*, *M. major*, *M. mangshanensis*, *M. maosonensis*, *M. megacephala*, *M. monticola*, *M. oreocrypta*, *M. oropedion*, *M. parva*, *M. periosa*, *M. serchhipii*, *M. takensis*, *M. zhangi*, and *M. zunhebotoensis* (vs. present in the latter).

By tips of fingers II-IV flat, expand to small pad, *Megophrys yeae* sp. nov. differs from Megophrys cf. pachyproctus, *Megophrys zhoui* sp. nov., *M. pachyproctus*, *M. acuta*, *M. binlingensis*, *M. brachykolos*, *M. cheni*, *M. lini*, *M. minor*, *M. obesa*, *M. palpebralespinosa*, *M. sangzhiensis*, *M. shuichengensis*, *M. spinata*, *M. tuberogranulata*, *M. wugongensis*, *M. mufumontana*, *M. auralensis*, and *M. robusta* (vs. expanded pads on fingertips absent in the latter).

By foot of males shorter (FOL 10.8–12.6 mm, n = 12), tympanum relatively smaller (males TD/EL 0.36–0.46, n = 12), and toes with narrow lateral fringes, *Megophrys yeae* sp. nov. further differs from *M. vegrandis* (vs. FOL 13.2–13.8 mm, n = 4, P < 0.001; TYD/EL 0.44–0.56, n = 4, P < 0.03; and fringes on toes wide in the latter).

By dorsal skin being relatively smooth, *Megophrys yeae* sp. nov. differs from *M. feii* (vs. dorsal skin rough in the latter).

*Megophrys yeae* sp. nov. differs from *M. medogensis* by the following characters: nuptial pad absent (vs. present in the latter); and base of first finger weak, size equal to the base of second finger, relative finger lengths I < II < IV < III (vs. base of finger I strong, larger than base of finger II, relative finger lengths II < I < IV < III in the latter).

By having following differences on skull morphology, *Megophrys yeae* sp. nov. differs from *M. medogensis*: skull weakly ossified, opening of anterior fontanelle large (vs. skull well ossified, opening of anterior fontanelle occlusive in the latter); premaxillary and maxillary teeth weak, separated from others by gaps (vs. strong, closely positioned with others in the latter); texture of sphenethmoid smooth, without curves and pits (vs. relatively smooth, with few pits in the latter); frontoparietal front equals rear (vs. distinctly wider in the latter); exoccipitals posterior to the line connecting conjunctions of quadratojugal and mandible (vs. anterior); and columella auris short (vs. long in the latter).

By having following differences on bioacoustics, *Megophrys yeae* sp. nov. differs from *M. medogensis*: dominant frequency significantly higher (4.4–5.2 kHz vs. 2.3–3.0 kHz in the latter; P < 0.001); call significantly faster (repetition rate average 3.0, vary from 1.9 to 4.1 vs. average 1.2 vary from 0.6 to 2.2 in the latter); and call intervals significantly longer (493–720 ms vs. 153–254 ms in the latter; P < 0.001).

By having the following characters, *Megophrys yeae* sp. nov. differs from *M. pachyproctus*: lacking a swollen protruding beyond cloaca (vs. present in the latter); nuptial pad absent (vs. present in the latter); and base of first finger weak, size equal to the base of second finger (vs. base of finger I strong, larger than base of finger II in the latter).

By having the following characters on skull morphology, *Megophrys yeae* sp. nov. differs from *M. pachyproctus*: premaxillary and maxillary teeth weak, separated from others by gaps (vs. strong, closely positioned with others in the latter); texture of sphenethmoid smooth, without curves and pits (vs. relatively smooth, with few pits in the latter); anterior fontanelle opening large (vs. occlusive in the latter); and sagittal suture occlusive (vs. distinctly open in the latter).

By having the following characters, *Megophrys yeae* sp. nov. differs from Megophrys cf. pachyproctus: nuptial pad absent (vs. present on finger I in the latter); and base of first finger weak, size equal to the base of second finger (vs. strong, larger than base of finger II in the latter).

By having following characters on skull morphology, *Megophrys yeae* sp. nov. differs from Megophrys cf. pachyproctus: texture of sphenethmoid smooth, without curves and pits (vs. rough, with curves and pits in the latter); anterior fontanelle opening large (vs. small, width equals sagittal suture in the latter); and sagittal suture occlusive (vs. distinctly open in the latter).

By having the following acoustical characters, *Megophrys yeae* sp. nov. differs from Megophrys cf. pachyproctus: call significantly shorter (99–212 ms, n = 6 vs. 491–889 ms, n = 3 in the latter; P < 0.001); dominant frequency much higher (4.4–5.2 kHz, n = 6 vs. 3.2–3.3 kHz, n = 3 in the latter; P < 0.001); call intervals significantly shorter (146–370 ms, n = 6, vs. 493–720 ms, n = 3 in the latter; P < 0.001); and calls significantly faster (call repetition rate1.9–4.1 call/s, n = 6, vs. 0.7–1.1call/s, n = 3 in the latter; P < 0.01).

By having following characters on skull morphology, *Megophrys yeae* sp. nov. differs from *Megophrys zhoui* sp. nov.: texture of sphenethmoid smooth, without curves and pits (vs. relatively smooth, with several small pits in the latter); and sagittal suture occlusive (vs. narrowly or wide open in the latter).

## Discussion

Similar to our surveys, only relatively few herpetologists have conducted field work in the eastern corner of Himalayas, mainly in Medog County, China (e.g., Huang and Fei 1981; Fei et al. 1983; Li et al. 2010; Jiang et al. 2012; Jiang et al. 2016a, b, and c). Several factors probably hindered the discoveries of the three new *Megophrys* species described here. First, in this region, *M. medogensis*, especially its tadpoles, are almost sympatric with all other related species’ tadpoles at extensive elevations even in the microhabitats, probably arousing the judgement of “one population with one species”. Moreover, the related species were superficially similar morphologically, easily misleading the identifications if made without detailed examination, especially for the first identification in the field. Of course, the third was insufficient expeditions. Chen et al. (2016) recognized two specimens KIZ010978 and KIZ011175 from Medog County as *M. pachyproctus* without reporting their morphological information. But in our phylogenetic trees, these two specimens were deeply nested into the *Megophrys yeae* sp. nov. clade (Fig. 2). Additionally, our results suggested that *Megophrys yeae* sp. nov. differs distinctly from *M. pachyproctus* on morphology (Figs 3, 5; Tables 1, 2). Hence, we propose that the two specimens were misidentified in this literature and that they should be classified as *Megophrys yeae* sp. nov. Similarly, Liu et al. (2018) treated one sample SYSa002934 from Medog County as *M. pachyproctus*. Our analyses, however, nested this sample into the *M. medogensis* clade (Fig. 2). *Megophrys pachyproctus* and *M. medogensis* should be classified as different species groups based on their morphology: the much larger body size of *M. medogensis* in the large-body-size clade (*M. major* complex proposed in Mahony et al. 2018), also indicating that the two species should be phylogenetically distinct. In any case, all these specimens should be reexamined.

By the protruding vent, *M. pachyproctus* differs from almost all species of *Megophrys* except *M. caudoprocta* and *M. koui*. The protruding vent of *M. caudoprocta* includes an elongated urostyle that slightly exceed ischium (Shen et al. 2013: fig. 1). However, the protruding vent of *M. pachyproctus* is a swelling and the urostyle does not exceed the vent. Furthermore, according to Yang and Rao (2008), the specimens of Megophrys (Ophryophyne) from the type locality (Zhushihe, Mengla, Yunnan Province, China) of *M. koui* vary in the presence of protruding vent while they share other morphological characters (identical skin ridge patterns and horn on outer edge of upper eyelid). Furthermore, *M. pachyproctus* was described based only on two males and one female. All these observations increase the uncertainty of whether the swelling protrusion can be used as a diagnostic character of *M. pachyproctus*. Our specimens M. cf. pachyproctus from Renqinbeng and Bari differ from the holotype of *M. pachyproctus* from Gelin mostly in the following characters: protuberance beyond cloaca small, barely visible from ventral view, not swollen (vs. protuberance present on vent beyond cloaca large, swollen, arc-shaped, visible on both dorsal and lateral view in the latter); and inner metatarsal tubercle distinct partially fused with toe I (vs. separate from base of toe I at a distance nearly twice its diameter in the latter). But M. cf. pachyproctus is similar to *M. pachyproctus* on many other morphological characters (e.g., body measurements, skin texture and skin ridges, and most characters on skull; Suppl. material 1: Table S5). For the moment, only one specimen (the holotype) of *M. pachyproctus* was examined, and there is no available molecular evidence from samples from Gelin; therefore, it is not prudent to erect a new name while there are still enigmas. Thus, we temporally treat these specimens from Renqingbeng and Bari as *M.*cf. pachyproctus. Further sampling at Gelin would help to resolve this problem in the future.

In this work, we classified samples of *M. medogensis* as low-middle-elevation group (682–1560 m) and high-elevation group (> 2100 m), because these samples phylogenetically clustered into two lineages based on mitochondrial DNA dataset but formed a single lineage when based on nuclear DNA dataset. The discordance indicates introgression between these two groups. The tadpoles of high-elevation group are morphologically different from the low-middle-elevation group: body coloration deep brown with copper pigmentation vs. body yellow-brown without copper pigmentation; tail muscle weaker (TMW/BW 44%) than the latter (TMW/BW 53–57%); lateral tail without dark patches vs. present. The morphological comparisons between adults of the two groups were not applicable in this work because no adults of the high-elevation groups were collected. The scenario of phylogenetical discordance between different gene datasets was also found in *M. monticola* (Mahony et al. 2018). The mechanism of two discovered cases of introgression from southeastern Himalayan is fascinating for further study. Note that the phylogenetically sister species of *Megophrys* in this region (i.e., *Megophrys yeae* sp. nov., *Megophrys zhoui* sp. nov., and *M. vegrandis* being genetically closer; Fig. 2) are distributed in different sites or altitudes, i.e., *Megophrys zhoui* sp. nov. just lives above 2000 m near the Renqingbeng Temple in Medog County, *Megophrys yeae* sp. nov. has a larger range but in some other sites at elevations between 500–1800 m (Figs 1, 10, and Suppl. material 2: Fig. S5) in Medog, and *M. vegrandis* has been just found at 1110 m in a southwestern locality away from the type localities of the first two relatives (Mahony et al. 2013). This case fits the “micro-endemism” model (Liu et al. 2018; Wang et al. 2019) for separating closely related species. On the other hand, the more “phylogenetically distant” species are often sympatric in microhabitat, such as tadpoles of Megophrys cf. pachyproctus and *Megophrys yeae* sp. nov. in the same pond, and *M. medogensis* with Megophrys cf. pachyproctus, *Megophrys zhoui* sp. nov., and *Megophrys yeae* sp. nov. in the same stream, indicating the “sympatric but phylogenetically distant” model. These biogeographical patterns have often been found in *Megophrys* (Mahony et al. 2018; Liu et al. 2018; Wang et al. 2019), indicating a complicated picture of biogeographical history of this taxonomically diverse toad group.

Separations of the horned toad species in Medog are also likely reflected on their different behaviors. Although being sympatric even in the same stream at elevations between 1500–1800 m in Bari village (Fig. 1), *M. medogensis* prefers tropical and subtropical forest floor (Fig. 10F), while *Megophrys yeae* sp. nov. is typically found calling on the leaves of tall dense plants (Fig. 10G), and Megophrys cf. pachyproctus calls on the branches of bushes (Fig. 10H). It is interesting that Megophrys cf. pachyproctus possess unique long calls, making a distinctly contrast with *Megophrys yeae* sp. nov. in the same stream which emit short calls (Fig. 4B, C; Table 3). The distinct calling patterns especially in the two “standing-upper” species probably prevent their calls overlapping in the upper space. This kind of “so-small-microhabitat” niche divergences may be also related with phenotype differences between them. The “floor” toad *M. medogensis* presents bigger body size, while the two “standing-on-plants or leaves” species have a pale body. Probably for further isolations, Megophrys cf. pachyproctus with relatively moderate body size prefers relative harder branches, vines, or stem of plants (Fig. 10H, I), while the sympatric species *Megophrys yeae* sp. nov. and *Megophrys zhoui* sp. nov. often stand on soft leaves and/or grass by their lighter body (Fig. 10G, J), even developing finger pads for climbing like tree frogs (Table 2; Fei et al. 2009). It is fascinating on exploring how their behaviors with corresponding morphological characteristics have been evolved to fitting corresponding environments.

The discoveries of the new species indicate a much-underestimated biodiversity in the Himalayan Mountains. Yet, the amphibians in the region are suffering from obvious threats in their habitats, for example, the ongoing construction of roads, towns, and houses, the use of pesticide chemicals for farming, and increasing activities of tourists. And, we also still have a poor understanding of the influences of local and/or global climatic changes. Undoubtedly, it is urgent to investigate their population status for the conservation of these extraordinary toads.

## Supplementary Material

XML Treatment for Megophrys pachyproctus

XML Treatment for Megophrys medogensis

XML Treatment for Megophrys cf. pachyproctus

XML Treatment for Megophrys zhoui

XML Treatment for Megophrys yeae

## Footnotes

For detailed comparisons, we re-described M. pachyproctus mainly based on the holotype NWIPB 770650 and added our skull information for it.

For detailed comparisons, we supplied morphology description of skull and tadpole for M. medogensis mainly based on the specimens collected in this study and added bioacoustics data for this species.

For detailed comparisons between these all related species, we independently described specimens of M. cf. pachyproctus in detail.

## Supplementary materials

Supplementary material 1 Table S1–S5 Data type measurements Explanation note **Table S1.** Measurements of the adult specimens of *Megophrys* used in this study. Unit in mm. See abbreviations for the morphological characters in Materials and methods section. **Table S2.** Measurements of the tadpole specimens of *Megophrys* used in this study. Unit in mm. See abbreviations for the morphological characters in Materials and methods section. **Table S3.** Measurements of advertisement call parameters for three *Megophrys* species in Medog. Values are given as mean (ranging). **Table S4.** Mean genetic distance under uncorrected *p*-distance model between *Megophrys* species based on 16S gene. **Table S5.** Morphological comparisons between the *Megophrys* species from the eastern Himalayas. Measurements in mm. See abbreviations for the morphological characters in Materials and methods section. https://binary.pensoft.net/file/466662This dataset is made available under the Open Database License (http://opendatacommons.org/licenses/odbl/1.0/). The Open Database License (ODbL) is a license agreement intended to allow users to freely share, modify, and use this Dataset while maintaining this same freedom for others, provided that the original source and author(s) are credited.Shengchao Shi, Meihua Zhang, Feng Xie, Jianping Jiang, Wulin Liu, Li Ding, Li Luan, Bin Wang

Supplementary material 2 Figure S1–S5 Data type multimedia Explanation note **Figure S1.***Megophrys medogensis* in life from Medog. **A**, **B** dorsal and ventral views of adult male CIB022017061404DD from Didong village, respectively **C**, **D** dorsal and ventral views of adult male CIB022017061405BB from Beibeng village, respectively **E** unvouchered calling male from Beibeng **F** adult female CIB022017061602 from Beibeng village. **Figure S2.** Photos showing variation of Megophrys cf. pachyproctus in life **A**, **B** adult male CIB022017061806 **C**, **D** adult male CIB022017061807 **E**, **F** gavid female CIBMT171054. Left: dorsolateral view; and right: ventral view. **Figure S3.** Photos of paratypes of *Megophrys zhoui* sp. nov. in life. **A**, **B** adult female CIBMT171062 **C**, **D** adult female CIBMT171060. Left for dorsolateral view, and right for ventral view. **Figure S4.** Photos of paratypes of *Megophrys yeae* sp. nov. in life **A**, **B** adult female CIB201706MT03 **C**, **D** adult male CIB022017061103 **E** adult male CIBMT171065 **F** adult male CIBMT171066. Left: dorsolateral view, and right: ventral view. **Figure S5.** Typical forests inhabited by *Megophrys* toads in Medog **A** landscape in Didong village at elevations of 600–850 m, harboring the low-middle-elevation *M. medogensis* and *Megophrys yeae* sp. nov. **B** landscape in Bari village and vicinity of Renqingbeng Temple at elevations of ca. 1400–2100 m, the former harboring low-middle-elevation *M. medogensis*, M. cf. pachyproctus, and *Megophrys yeae* sp. nov., and the latter hosting M. cf. pachyproctus and *Megophrys zhoui* sp. nov. **C** landscape in Gelin village at elevations of ca. 1500–1800 m, hosting the low-middle-elevation *M. medogensis* and *M. pachyproctus***D** Gedang village at elevations above 2100 m, harboring the high-elevation *M. medogensis***E**–**H** local forests in the four sites, respectively. https://binary.pensoft.net/file/466663This dataset is made available under the Open Database License (http://opendatacommons.org/licenses/odbl/1.0/). The Open Database License (ODbL) is a license agreement intended to allow users to freely share, modify, and use this Dataset while maintaining this same freedom for others, provided that the original source and author(s) are credited.Shengchao Shi, Meihua Zhang, Feng Xie, Jianping Jiang, Wulin Liu, Li Ding, Li Luan, Bin Wang
