# Supplementary figures and images for: Multiple data revealed two new species of the Asian horned toad Megophrys Kuhl & Van Hasselt, 1822 (Anura, Megophryidae) from the eastern corner of the Himalayas
Source: Zookeys. 2020 Oct 22;977:101–61. doi: 10.3897/zookeys.977.55693 (PMC7596021; doi:10.3897/zookeys.977.55693)

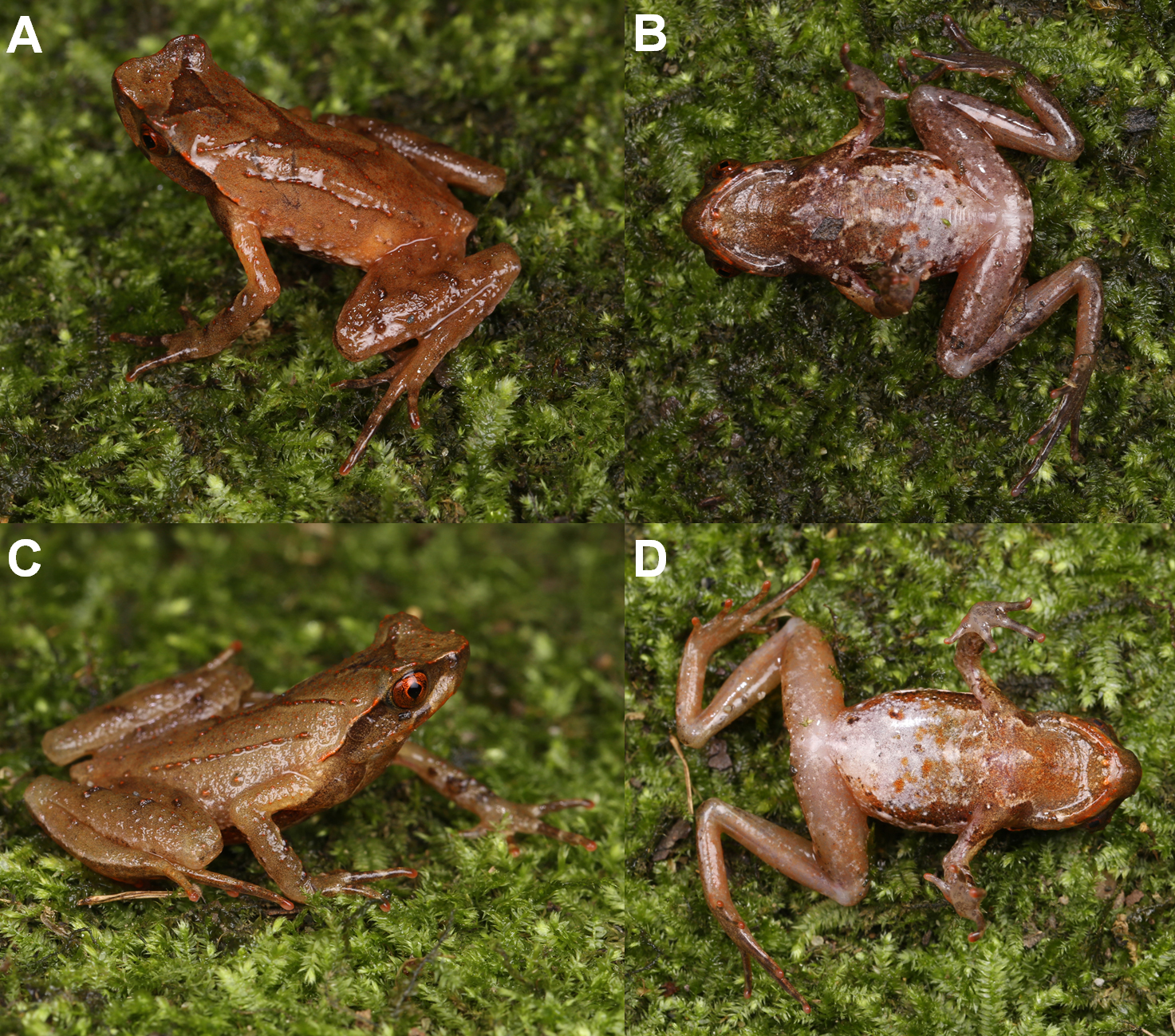

Supplement: Supplementary material 2 — Figure S1–S5 [file zookeys-977-101-s002.zip › 55693_0R-2-A_revised Figure S3.jpg]

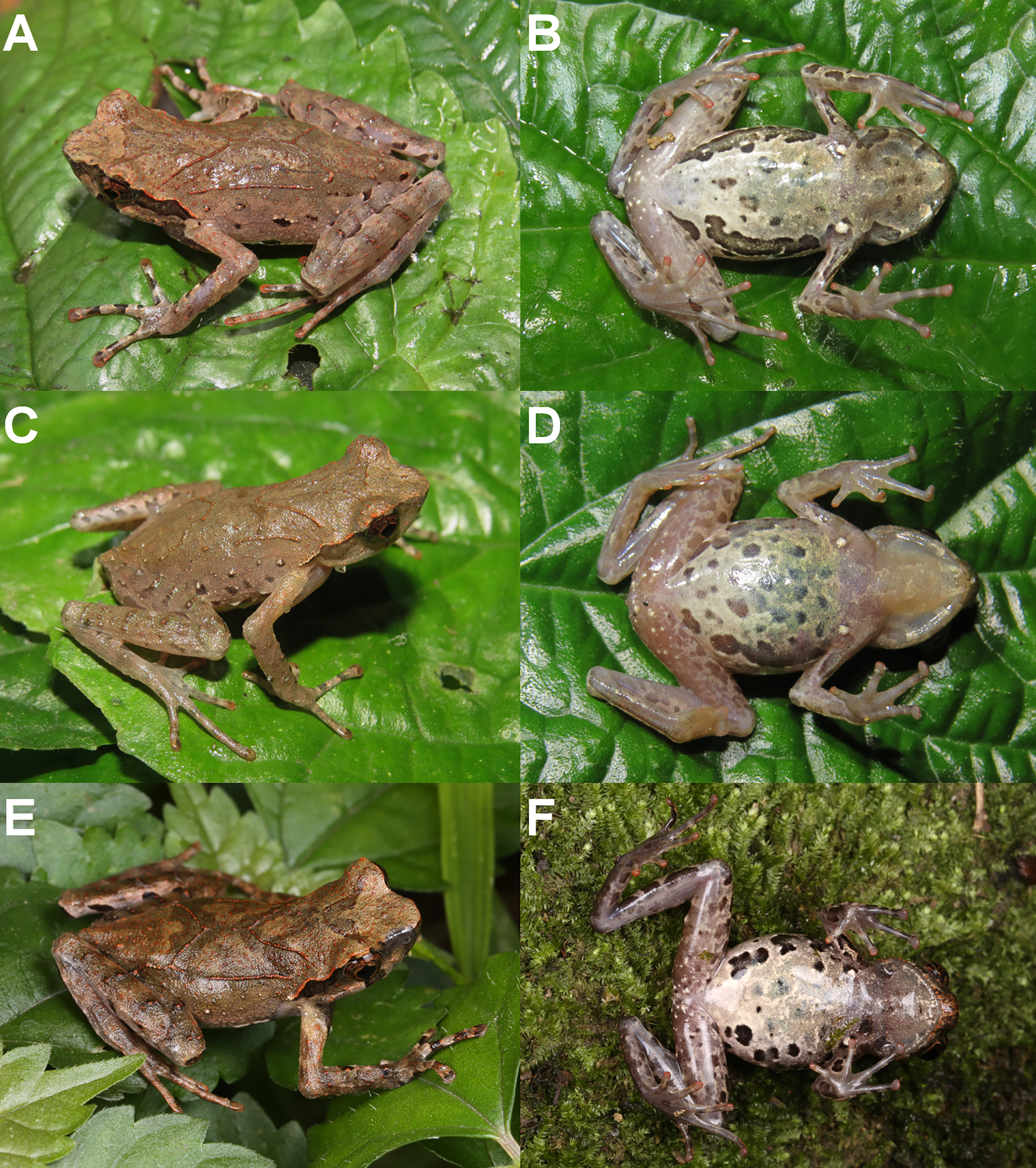

Supplement: Supplementary material 2 — Figure S1–S5 [file zookeys-977-101-s002.zip › 55693_0R-2-A_revised Figure S4.jpg]

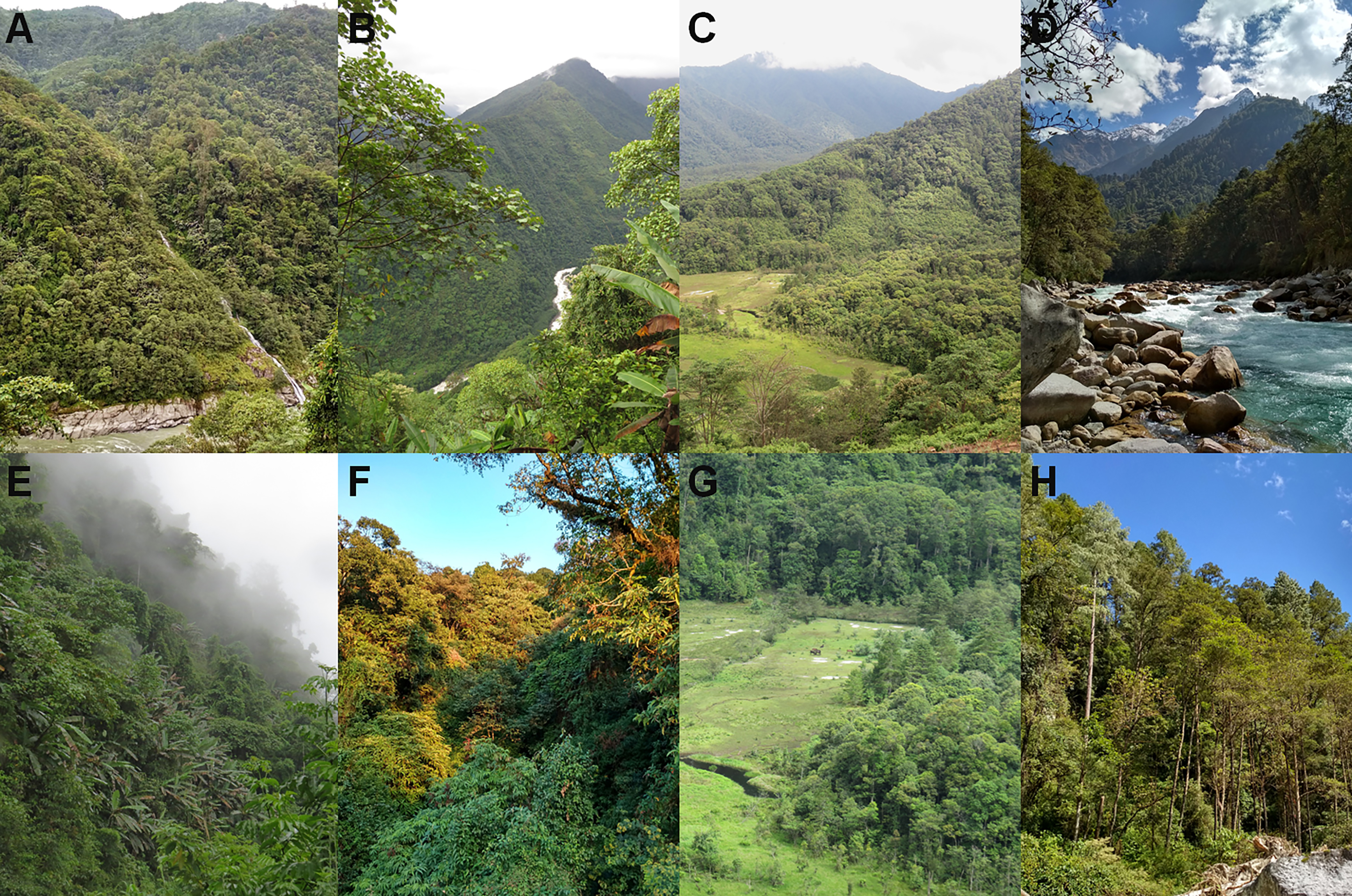

Supplement: Supplementary material 2 — Figure S1–S5 [file zookeys-977-101-s002.zip › 55693_0R-2-A_revised Figure S5.jpg]

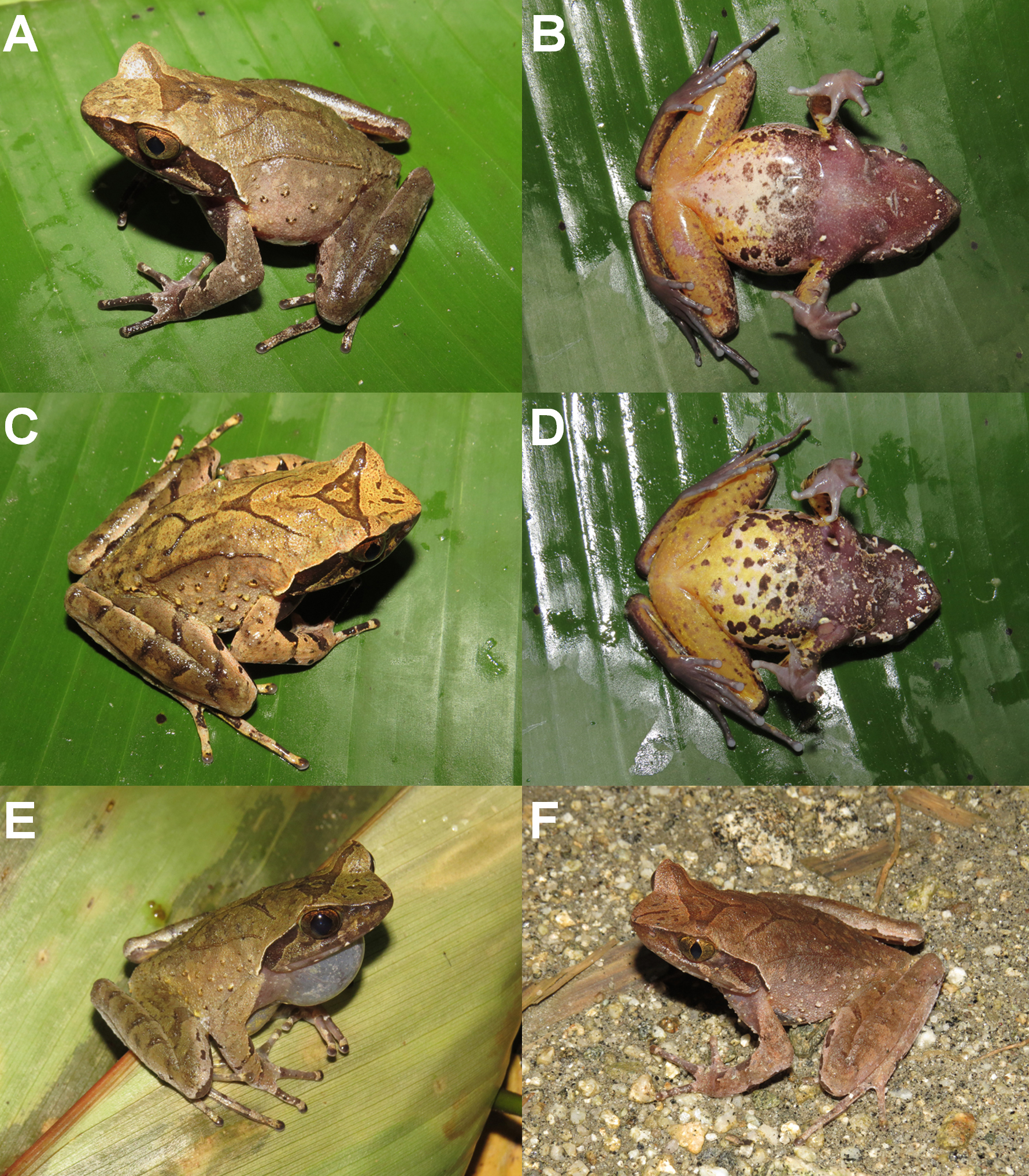

Supplement: Supplementary material 2 — Figure S1–S5 [file zookeys-977-101-s002.zip › 55693_0R-2-A_revised Figure S1.jpg]

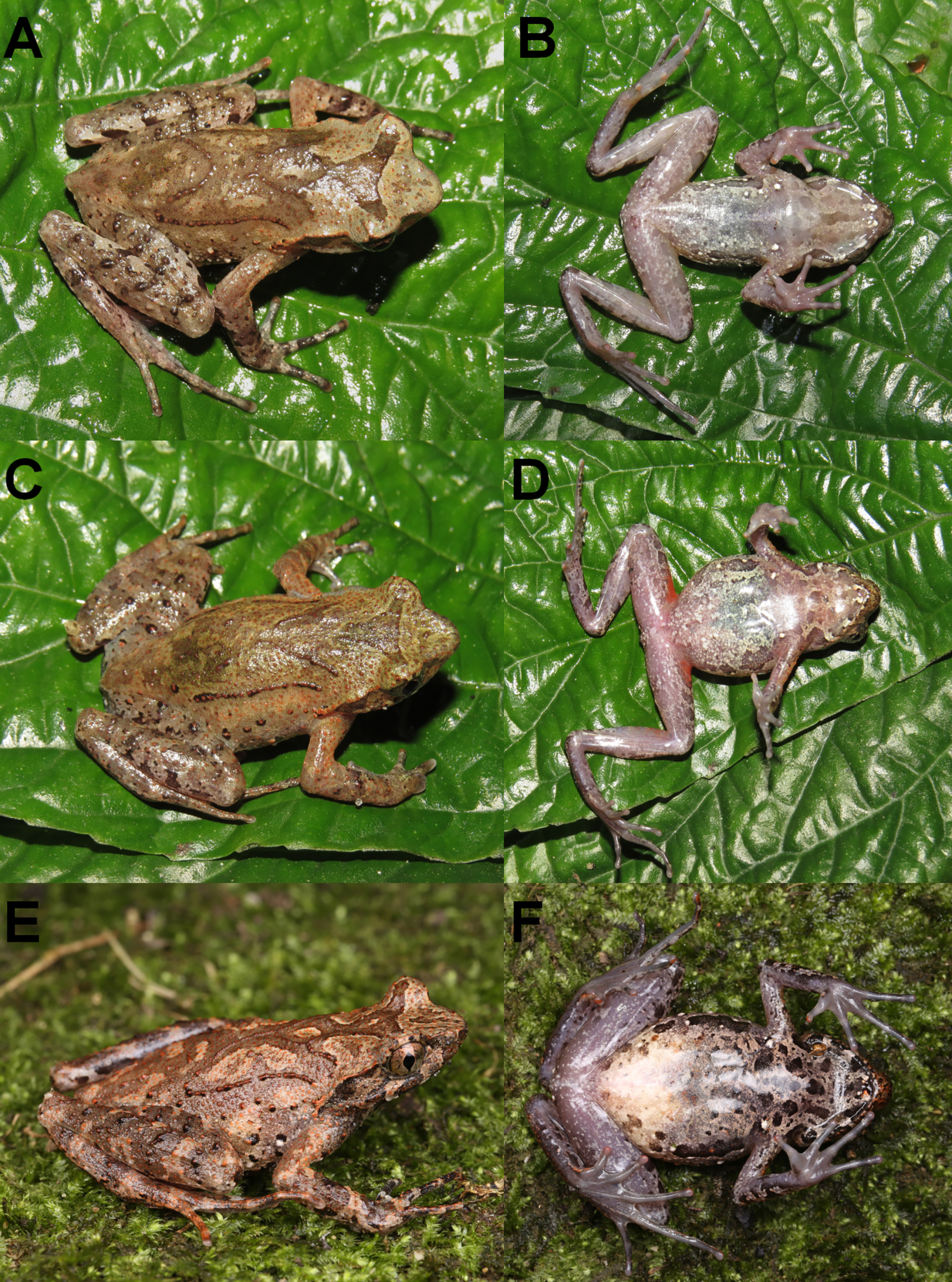

Supplement: Supplementary material 2 — Figure S1–S5 [file zookeys-977-101-s002.zip › 55693_0R-2-A_revised Figure S2.jpg]
